# Type VII secretion system deploys an active iron uptake pathway to enhance bacterial fitness and counteract host nutritional immunity

**DOI:** 10.1128/mbio.02419-25

**Published:** 2025-09-25

**Authors:** Chunhui Luo, Xinwei Hao, Xiao Wang, Mohua Liu, Shukun Chen, Conghui Wu, Chen Li, Can Chen, Mingming Yang, Lei Xu, Yao Wang, Xihui Shen

**Affiliations:** 1 Shaanxi Key Laboratory of Agricultural and Environmental Microbiology, College of Life Sciences, Northwest A&F University 12469, Yangling, Shaanxi, China; 2 Jiangsu Key Laboratory of Marine Bioresources and Environment, College of Marine Food and Bioengineering, Jiangsu Ocean University 66486, Lianyungang, Jiangsu, China; 3 Key Laboratory of Plant Genetics and Molecular Breeding, Henan Key Laboratory of Crop Molecular Breeding and Bioreactor, School of Life Sciences and Agronomy, Zhoukou Normal University 117778, Zhoukou, China; 4 College of Plant Protection, Northwest A&F University 12469, Yangling, Shaanxi, China; Georgia Institute of Technology, Atlanta, Georgia, USA

**Keywords:** type VII secretion system (T7SS), iron acquisition, oxidative stress, nutritional immunity, host–pathogen interactions

## Abstract

Type VII secretion systems (T7SS) are increasingly recognized as pivotal for bacterial adaptation across both environmental and host-associated niches in Gram-positive bacteria. Although T7SS has been reported to contribute to iron acquisition, the possibility of an active, more targeted iron uptake mechanism remains underexplored. Here, we reveal a distinct, active T7SS-mediated iron import mechanism in *Corynebacterium glutamicum*. We show that T7SS expression is induced under iron limitation and oxidative stress conditions and is tightly regulated by the ferric uptake regulator (Fur) to benefit bacterial survival. Surprisingly, this iron uptake pathway relies on the T7SS-secreted effector ExsI, an iron-binding protein that collaborates with the membrane receptor ExiR to facilitate iron uptake into the cytoplasm. This ExsI-ExiR-mediated active iron acquisition mechanism enhances bacterial oxidative stress resistance and confers a competitive advantage in iron-limited environments. Furthermore, we show that ExsI homologs in *Mycobacterium smegmatis* circumvent calprotectin-mediated nutritional immunity, extending the relevance of this mechanism to host–pathogen interactions. Taken together, our data demonstrate a key role for T7SS in orchestrating active iron uptake, oxidative stress resilience, competitive fitness, and evasion of host nutritional immunity, highlighting its significant role in bacterial physiology and host–pathogen interactions.

**IMPORTANCE:**

Type VII secretion systems (T7SS) are increasingly recognized as an indispensable secretion system for Gram-positive bacteria, mediating processes vital for bacterial survival and pathogenesis. This work reveals a previously unrecognized mode of active iron acquisition mediated by T7SS, which not only boosts oxidative stress resistance in *Corynebacterium glutamicum* but also provides a competitive edge under nutrient-limited conditions. ExsI homologs in *Mycobacterium smegmatis* overcome calprotectin-mediated iron withholding, suggesting that T7SS-driven iron acquisition extends to immune evasion. These uncovered previously unreported functions of T7SS emphasize the indispensable importance of T7SS in bacterial physiology, enhancing our understanding of T7SS.

## INTRODUCTION

Bacteria have evolved sophisticated and tightly regulated secretion systems to survive and thrive in diverse settings that facilitate interactions with their surroundings, neighboring microorganisms, and host organisms. Among these, the type VII secretion system (T7SS), also known as the ESAT-6 secretion (ESX) system, has emerged as a critical mechanism in Gram-positive bacteria (1). A defining feature of the T7SS is its ESX secretion machinery, closely associated with the WXG100 protein family, as well as the proline-glutamic acid (PE) and proline-proline-glutamic acid (PPE) families of effectors (2). Protein effectors transport within the ESX system is powered by substrate-specific ATPases, with core components EccB, EccD, EccE, and EccC forming a stable multi-protein complex in the mycobacterial inner membrane (1, 3). This multi-protein complex is the foundation for the secretion machinery, ensuring the coordinated translocation of effector molecules (1, 4, 5).

Initially identified in *Mycobacterium tuberculosis*, the causative agent of tuberculosis, T7SS has since been recognized as a vital determinant of virulence and survival across a broad spectrum of Gram-positive bacteria (2). T7SS has been reported to be involved in various biological processes, including nutrient acquisition, DNA transfer, and bacterial competition, underscoring its multifunctional role in bacterial physiology (6–11). T7SS garnered attention for secreting key virulence factors, such as ESAT-6 (EsxA) and CFP-10 (EsxB), which enable *M. tuberculosis* to evade host immune defenses and establish persistent infections (12, 13). Subsequent research revealed its critical role in the survival and pathogenicity of various mycobacteria and related actinobacteria (14, 15).

A particularly significant function of T7SS is its involvement in iron homeostasis and acquisition (16–20). Iron is an indispensable micronutrient for bacteria, serving as a cofactor in numerous enzymatic processes vital for DNA synthesis, respiration, and defense against oxidative stress (21–25). In bacterial cells, iron contributes to oxidative stress resistance by acting as a cofactor for enzymes that detoxify reactive oxygen species (ROS) and support antioxidant defenses (24–27). However, iron’s bioavailability is limited in aerobic environments due to the low solubility of its ferric (Fe^3+^) form. To overcome this challenge, bacteria have developed sophisticated iron uptake systems, including direct uptake and siderophore-mediated mechanisms (28–31). Siderophores are high-affinity iron-chelating molecules that scavenge iron from the environment and facilitate its transport into the cell (28, 29). In particular, mycobacteria produce salicylate-containing siderophores known as mycobactins, and *Mycobacterium smegmatis* additionally secretes peptide-based siderophores named exochelins (30, 31).

Recent studies have showcased a previously underexplored role of the T7SS in iron acquisition (32). In *M. tuberculosis*, for instance, the ESX-3 locus, one of five characterized T7SS loci (ESX-1 to ESX-5), facilitates iron uptake from mycobactins, while ESX-4 mediates extracellular heme uptake (20, 32, 33). These processes largely rely on mycobacterial PE-PPE protein complexes, which function as channels that import iron-bearing molecules (34, 35), thus requiring a sufficiently high external concentration of iron carriers. In addition, in the plant-beneficial *Bacillus velezensis* SQR9, a T7SS-secreted protein (YukE) actively alters the local environment by triggering iron leakage from plant roots that promotes its root colonization, expanding T7SS’s known repertoire for managing iron scarcity (36). These studies highlight the vital role of iron bioavailability and acquisition in shaping bacterial viability, community dynamics, and host-microbe interactions, prompting further investigation into the mechanistic intricacies of T7SS-mediated iron transport. However, whether the T7SS-secreted effector is involved in a more active iron uptake mechanism remains unclear and needs further exploration.

Iron acquisition strategies are further complicated by host-imposed nutritional immunity, a defense mechanism wherein host organisms limit the availability of essential metal ions to invading pathogens (37, 38). Proteins such as calprotectin (CP), composed of the S100A8 and S100A9 subunits, play a central role in this process by sequestering vital metals like manganese (Mn^2+^) and zinc (Zn^2+^), and recent studies have shown that CP also binds ferric iron (Fe^3+^) (39, 40). Meanwhile, many bacteria have developed sophisticated strategies to circumvent host-imposed nutritional immunity (37, 41), including the production of high-affinity iron chelators like siderophores to scavenge essential metals. However, the role of the T7SS-mediated metal ion acquisition in countering CP-mediated nutritional immunity remains poorly understood.

In this study, *Corynebacterium glutamicum* was used as a model organism for dissecting the mechanistic roles of T7SS in iron acquisition. Unlike other bacteria that harbor multiple T7SS loci, non-pathogenic *C. glutamicum* contains a single T7SS locus (1, 42). This single T7SS locus is homologous to the ESX-4 in *M. tuberculosis*, which is considered the ancestral ESX secretion system that harbors fewer genes than other *esx* loci and is conserved across all mycobacterial species (1, 43). This simplicity makes *C. glutamicum* an excellent platform for studying T7SS function in isolation. We found that *C. glutamicum* T7SS secretes an iron-binding effector, ExsI (Esx-secreted iron-binding effector), to capture free iron from the environment by interacting with a membrane receptor ExiR (Esx-secreted iron-binding effector receptor). Additionally, an ExsI homolog (ExsI^ms^) in *M. smegmatis* enhances bacterial survival by counteracting host CP-mediated nutritional immunity. We demonstrated that this iron-scavenging effector expands the functional repertoire of the *C. glutamicum* T7SS by mediating oxidative stress resistance, exploitative competition, and its homolog in *M. smegmatis* contributes to virulence by counteracting host nutritional immunity.

## RESULTS

### The Fur-regulated T7SS contributes to iron acquisition in *C. glutamicum*

**Fig 1:**
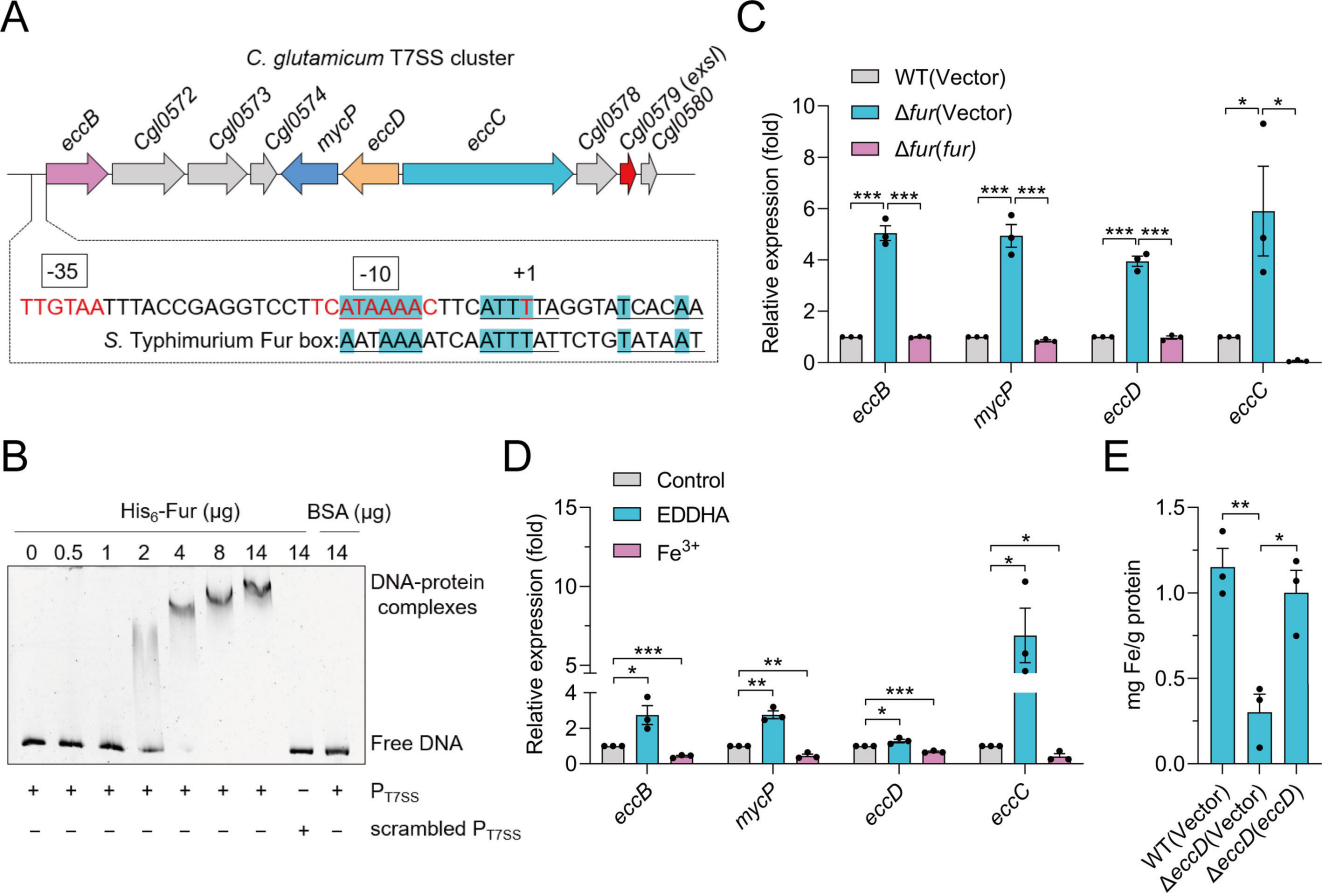
The Fur-regulated T7SS contributes to iron acquisition in *C. glutamicum*. (**A**) Schematic representation of the *C. glutamicum* genes encoding the T7SS cluster. The putative Fur-binding site in the promoter region of the T7SS gene cluster was identified using the online tool VirtualFootprint (https://www.prodoric.de/vfp), with the predicted binding site highlighted in blue. The putative −35 and −10 elements of the T7SS promoter were boxed. +1 denotes the transcription start point. (**B**) EMSA was performed to analyze the interactions between purified His_6_-Fur and the T7SS promoter. The increasing amount of purified His_6_-Fur ranged from 0 to 14 µg. The T7SS promoter probe was 90 ng (+177 to −173). A total of 14 µg of BSA protein and 90 ng of scrambled P_T7SS_ were included in the assay as negative controls. Representative images from three independent experiments. (**C**) The mRNA expression levels of *eccB*, *mycP*, *eccC,* and *eccD* in *C. glutamicum* WT, Δ*fur*, and Δ*fur*(*fur*) strains were measured by quantitative Reverse transcription PCR (qRT-PCR). (**D**) *C. glutamicum* WT strain was grown in LB medium containing 100 μM ethylenediamine-N,N′-bis(2-hydroxyphenylacetic acid) (EDDHA) or 100 μM Fe^3+^ to mid-exponential phase, and the expression of *eccB*, *mycP*, *eccC,* and *eccD* genes was measured by qRT-PCR. (**E**) Relevant late-exponential phase *C. glutamicum* strains were exposed in an M9 medium containing 15 mM H_2_O_2_ with the addition of 1 µM Fe^3+^ for 20 min. Fe associated with bacterial cells was measured by atomic absorption spectrometry analysis. (**C–E**) Data are mean ± SEM of three independent experiments. *P* values calculated using two-tailed Student’s *t*-test for paired comparisons or one-way analysis of variance (ANOVA) with Tukey’s multiple comparisons test. *P < 0.05; **P < 0.01; ***P < 0.001; ns, not significant.

*Corynebacterium glutamicum* ATCC13032 encodes only one *esx* locus, making it an ideal model organism for exploring the mechanistic role of T7SS(1, 42) (Fig. 1A). Analysis of the promoter region of the T7SS operon in *C. glutamicum* identified a potential binding site for the ferric uptake regulator protein (Fur), a well-known regulator for iron homeostasis (21), located upstream of the *eccB* gene (Fig. 1A). Notably, the identified Fur-binding site closely resembles the Fur-binding box found in *Salmonella* Typhimurium (Fig. 1A). Additionally, the amino acid sequence of Fur in *C. glutamicum* is similar to that in *Escherichia coli*, *Salmonella* Typhimurium, *M. smegmatis*, and *M. tuberculosis* (Fig. S1). To confirm Fur binding to the *T7SS* promoter, we performed an electrophoretic mobility shift assay (EMSA) with purified Fur protein and a DNA probe containing the T7SS promoter sequence (*P_t7ss_*). A dose-dependent shift in the mobility of the T7SS promoter probe was observed with increasing concentrations of Fur protein (Fig. 1B). To further investigate Fur’s regulatory role, we analyzed the transcription of T7SS-related genes in a *fur* deletion mutant (Δ*fur*). In the absence of Fur, the expression levels of *eccB* (*Cgl0571*), *mycP* (*Cgl0575*), *eccC* (*Cgl0577*), and *eccD* (*Cgl0576*) were significantly upregulated compared to the WT strain, and such a phenotype can be restored by Fur complementation (Fig. 1C). These findings indicate that Fur acts as a repressor of T7SS gene expression under normal conditions, likely in response to iron availability.

Given the role of Fur as an iron-dependent regulator (21, 44), we examined the expression of T7SS genes under iron-limited and iron-rich conditions. Excess iron inhibited T7SS operon expression, whereas chelating iron from the medium with EDDHA induced T7SS expression (Fig. 1D), suggesting T7SS may respond to extracellular iron levels. Next, a T7SS-deficient mutant was generated by deleting the *eccD* gene (encoding an integral membrane protein of the T7SS), and the intracellular iron content was measured. Atomic absorption spectrometry analysis revealed that the Δ*eccD* mutant that lacks the core membrane channel-forming protein EccD required for system assembly accumulated significantly less intracellular iron than the WT strain, with iron levels being restored in the complemented strain (Fig. 1E). These findings indicate that the T7SS is involved in iron acquisition and that its expression is regulated by iron availability. In summary, we demonstrated that the T7SS in *C. glutamicum* is directly regulated by Fur and is critical for efficient iron acquisition.

### *C. glutamicum* T7SS contributes to oxidative stress resistance by importing iron

Iron is a vital cofactor for enzymes involved in oxidative stress responses, and its bioavailability is essential for bacterial survival under oxidative stress (24). In *C. glutamicum*, hydrogen peroxide (H_2_O_2_) exposure markedly upregulated T7SS-associated genes (*eccB*, *eccC*, *eccD*, and *mycP*) (Fig. S2A), indicating T7SS activation during oxidative stress. To functionally investigate the role of T7SS in oxidative stress resistance in *C. glutamicum*, we examined a Δ*eccC* mutant deficient in the core T7SS ATPase EccC, which provides energy for effector secretion. The mutant displayed heightened sensitivity to H_2_O_2_ compared to wild-type cells, a phenotype that was fully rescued by *eccC* complementation (Fig. S2B). Oxidative stress is characterized by the accumulation of ROS (45). We found that the Δ*eccC* mutant exhibited significantly higher ROS accumulation than both wild-type and complemented strains after H_2_O_2_ challenge (Fig. S2C). Together, these findings demonstrate that T7SS enhances ROS detoxification in *C. glutamicum*, thereby mitigating oxidative damage.

**Fig 2:**
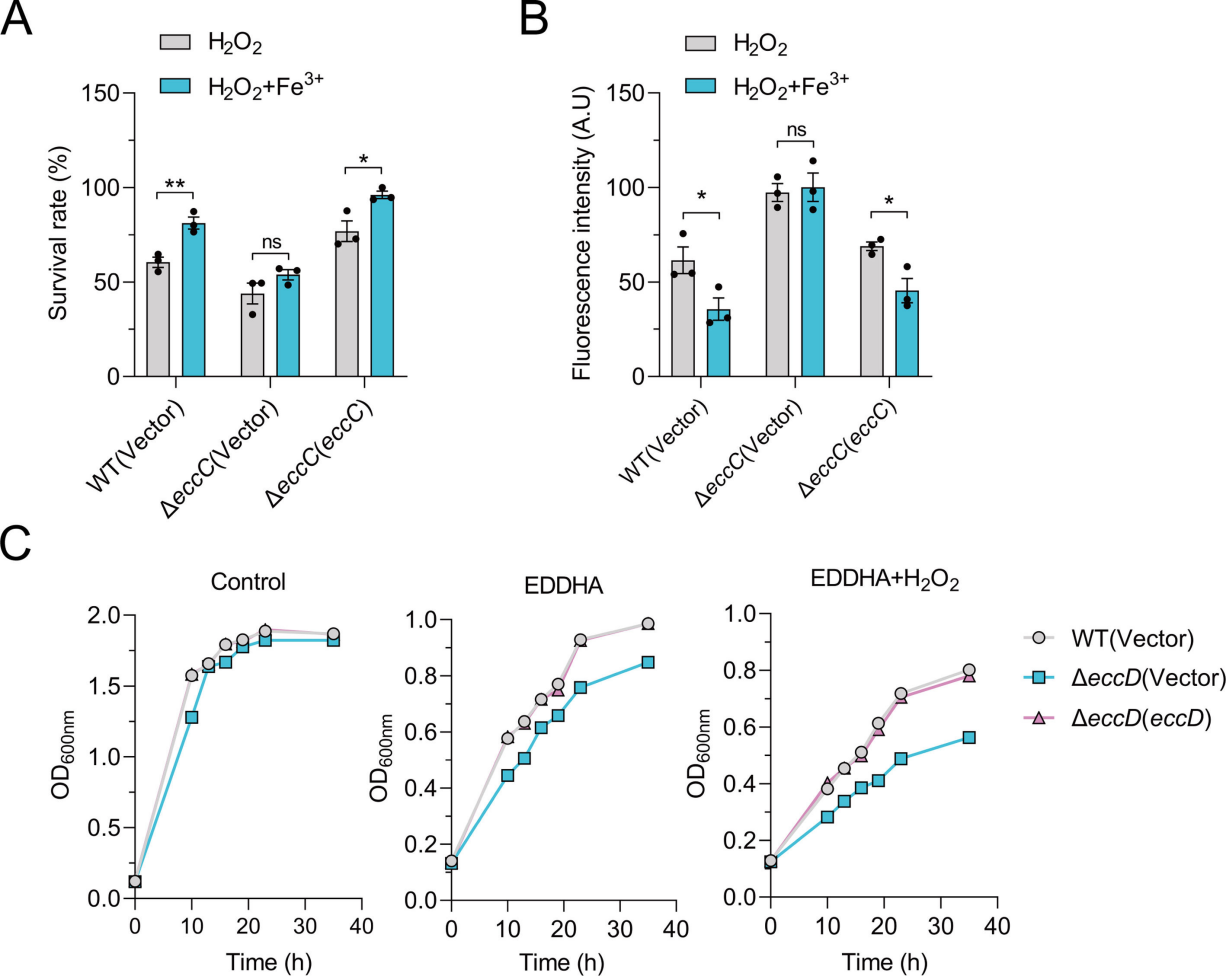
*C. glutamicum* T7SS imports iron to combat oxidative stress. (**A**) Relevant late-exponential phase bacterial strains were cultured in an M9 medium containing 15 mM H_2_O_2_ with or without the addition of Fe^3+^ (1 µM) for 40 min, and the viability of the bacterial cells was determined by spreading to agar plates with appropriate dilution. (**B**) Relevant late-exponential phase bacterial strains were cultured in an M9 medium containing 15 mM H_2_O_2_ with or without the addition of Fe^3+^ (1 µM) for 40 min, and the intracellular ROS levels were assessed using CM-H2DCFDA. The fluorescence signals were quantified with a SpectraMax M2 Plate Reader. (**C**) The related *C. glutamicum* strains grown overnight were harvested and diluted 100-fold into different media: LB medium (left), LB medium supplemented with 70 µM EDDHA (middle), and LB medium containing 70 µM EDDHA and 15 mM H_2_O_2_ (right). The cultures were incubated at 30°C, and their growth was monitored by measuring the OD_600_ at specified time points. Data in all panels are mean ± SEM of three independent experiments. *P* values were calculated using a two-tailed Student’s *t*-test for paired comparisons or one-way ANOVA with Tukey’s multiple comparisons test. *P < 0.05; **P < 0.01; ns, not significant.

To determine whether T7SS-mediated oxidative stress resistance depends on iron import, we tested bacterial survival and ROS levels upon iron supplementation during H_2_O_2_ challenge. While iron significantly improved survival and reduced ROS in WT and complemented strains, the Δ*eccC* mutant remained sensitive (Fig. 2A and B), suggesting T7SS is required for iron-dependent protection. We next assessed the role of iron import via T7SS under oxidative stress by measuring bacterial growth under iron-chelated (EDDHA) and oxidative stress conditions. Although growth in LB was unaffected, the Δ*eccD* mutant exhibited a growth defect under iron limitation, which worsened with combined H_2_O_2_ and EDDHA treatment (Fig. 2C). These results confirm the importance of T7SS-dependent iron import for bacterial survival under oxidative stress.

Microbial communities often experience competition for scarce nutrients like iron (46). The above results raised the question of whether the iron import capabilities mediated by T7SS could also influence the competitive dynamics of *C. glutamicum* in nutrient-limited environments. In iron-limited co-cultures with *Burkholderia thailandensis* E264 and *Escherichia coli* BL21, the Δ*eccD* mutant showed significantly reduced competitiveness versus WT and complemented strains (Fig. S3A). Crucially, iron supplementation eliminated WT’s competitive edge (Fig. S3B), further supporting the role of T7SS-mediated iron import in bacterial competition. These results establish T7SS as an important factor of C. *glutamicum*’s fitness in iron-limited environments.

### *C. glutamicum* T7SS secretes an iron-binding effector ExsI to facilitate iron import

Effective metal ion uptake systems often rely on the secretion of specialized metalloprotein effectors that facilitate the capture and transport of essential metals. We explored whether the T7SS in *C. glutamicum* might employ a similar mechanism. To this end, we focused on the WXG100 family proteins, which are typically secreted as ESAT-6-like proteins by the T7SS (Fig. 1A) (5, 13, 47). We identified two genes, *Cgl0579* and *Cgl0580*, located downstream of the T7SS core gene cluster, encoding WXG100 family proteins. Structural analysis of these proteins using Alphafold3 (48) and docking simulations was performed to analyze the interaction between protein models and Fe^3+^. The 3D structures showed that the Fe atom is wrapped by Cgl0579 but showed no interaction with Cgl0580, which suggests that Cgl0579 binds with Fe^3+^, while Cgl0580 could not (Fig. S4A). Supportively, the best docking conformation obtained by AutoDock Vina 1.2.5 (49, 50) suggests that the binding between Fe^3+^ and Cgl0579 is stronger than the binding between Fe^3+^ and Cgl0580, as indicated by the lower Δ*G* for the Cgl0579-Fe^3+^ (–4.88 kJ/mol) interaction compared to the Cgl0580-Fe^3+^ (–4.03 kJ/mol) interaction (Fig. S4A). Further structural modeling with I-TASSER predicted potential Fe^3+^-binding residues Glu8 and Met12 in Cgl0579 (Fig. S4B) (51). These *in silico* predictions suggest that Cgl0579 may function as an iron-binding protein.

**Fig 3:**
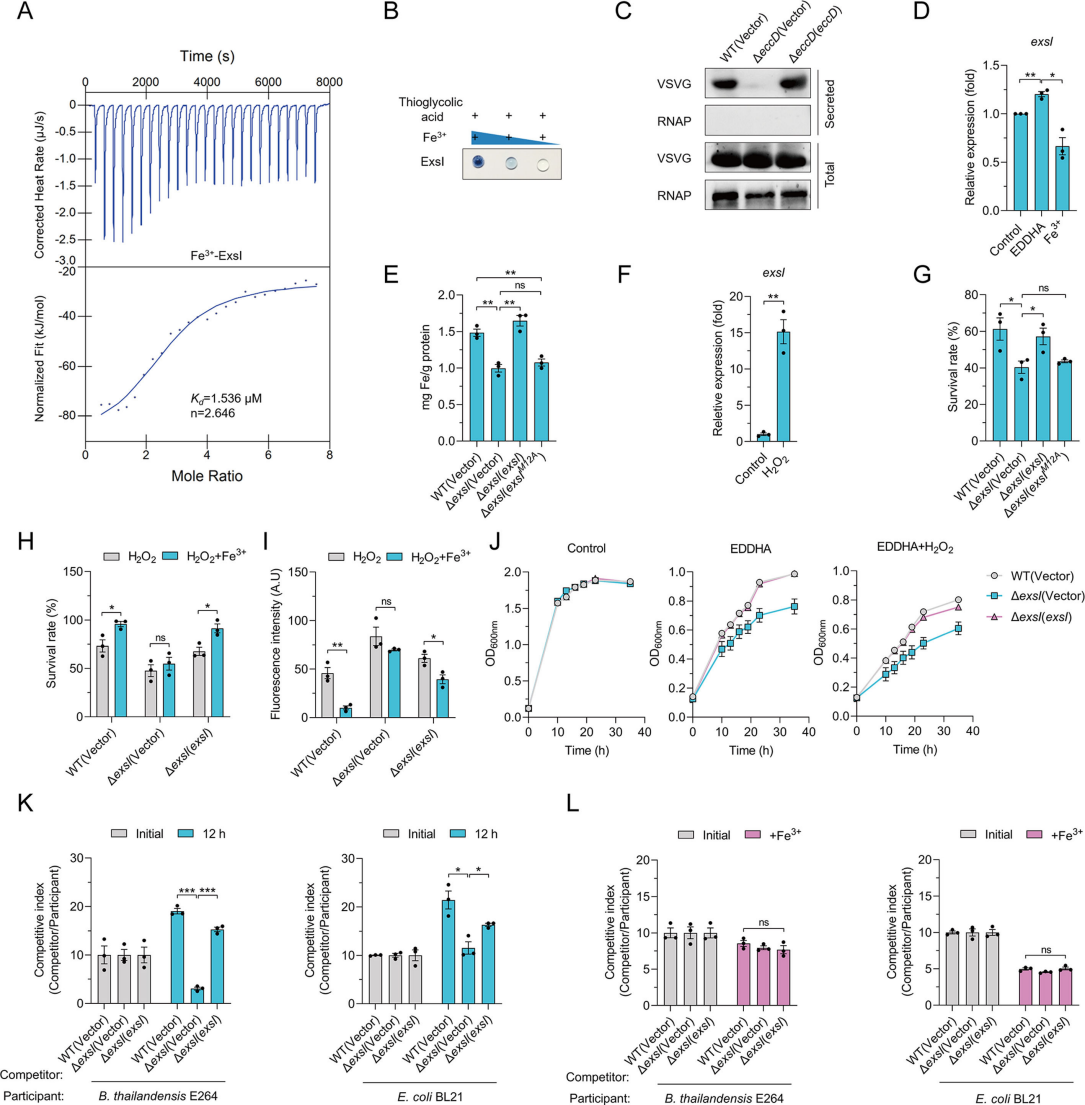
The T7SS secretes the iron-binding effector ExsI to facilitate iron import. (**A**) The binding of Fe^3+^ by ExsI. Metal ion-free ExsI was used to evaluate Fe^3+^-binding activity by ITC. Data were analyzed using the Nano Analyze software (TA Instruments). (**B**) The ability of ExsI to bind Fe^3+^ was assessed using a Ferene S staining assay. The ExsI protein was mixed with the stain, dot-blotted onto a nitrocellulose membrane, and then observed for the development of a blue color. (**C**) Different *C. glutamicum* strains expressing ExsI-VSVG were cultured until the late-exponential phase. ExsI-VSVG proteins in the total cell pellet (total) and the culture supernatant (secreted) samples were then detected by western blot. Cytosolic RNA polymerase (RNAP) was used as a control. (**D**) *C. glutamicum* WT strain was grown in LB medium containing 100 μM EDDHA or 100 μM Fe^3+^ to mid-exponential phase, and the expression of *exsI* was measured by qRT-PCR. (**E**) Relevant late-exponential phase bacterial strains were exposed in M9 medium containing 15 mM H_2_O_2_ with the addition of Fe^3+^ (1 µM) for 20 min. Iron associated with bacterial cells was measured by atomic absorption spectrometry analysis. (**F**) *C. glutamicum* WT strain was grown in LB medium and then exposed to 15 mM H_2_O_2_ for 5 min, and the mRNA expression of *exsI* was measured by qRT-PCR. (**G**) Relevant late-exponential phase bacterial strains were cultured in an M9 medium containing 15 mM H_2_O_2_ for 40 min, and the viability of the cells was determined. (**H**) Relevant late-exponential phase bacterial strains were cultured in M9 medium containing 15 mM H_2_O_2_ with the addition of Fe^3+^ (1 µM) for 40 min, and the viability of the cells was determined. (**I**) Relevant late-exponential phase bacterial strains were cultured in M9 medium containing 15 mM H_2_O_2_ with or without the addition of Fe^3+^ (1 µM) for 40 min, and the intracellular ROS levels were assessed using CM-H2DCFDA. The fluorescence signals were quantified with a SpectraMax M2 Plate Reader. (**J**) The related *C. glutamicum* strains grown overnight were harvested and diluted 100-fold into different media: LB medium (left), LB medium with 70 µM EDDHA (middle), and LB medium with 70 µM EDDHA and 15 mM H_2_O_2_ (right). The growth of the relevant strains was detected by measuring OD_600_ at the specified time points. (**K**) Bacterial competition between *C. glutamicum* and *B. thailandensis* or *E*. *coli*. *C. glutamicum* (competitor) was mixed with *B. thailandensis* (participant) in M9 medium in a ratio of 10:1 and grown at 30°C for 12 h (left). *C. glutamicum* (competitor) was mixed with *E. coli* (participant) in M9 medium in a ratio of 10:1 and grown at 26°C for 12 h (right). Bars represent the mean competitor: participant colony-forming units (CFU) ratios of three independent experiments (±SEM). (**L**) Bacterial competition between *C. glutamicum* and *B. thailandensis* or *E. coli. C. glutamicum* (competitor) was mixed with *B. thailandensis* (participant) in M9 medium containing 1 µM Fe^3+^ in a ratio of 10:1 and grown at 30°C for 12 h (left). *C. glutamicum* (competitor) was mixed with *E. coli* (participant) in M9 medium containing 1 µM Fe^3+^ in a ratio of 10:1 and grown at 26°C for 12 h (right). Bars represent the mean competitor: participant CFU ratios of three independent experiments (±SEM). (**A–C**) Representative images from three independent experiments. (**D–L**) Data are mean ± SEM of three independent experiments. *P* values were calculated using a two-tailed Student’s *t*-test for paired comparisons or one-way ANOVA with Tukey’s multiple comparisons test. *P < 0.05; **P < 0.01; ***P < 0.001; ns, not significant.

Experimental validation using isothermal titration calorimetry (ITC) confirmed that Cgl0579 binds Fe^3+^ with high specificity (dissociation constant [*Kd*] = 1.536 µM), whereas no binding was observed for Ca^2+^, Mg^2+^, or Mn^2+^ (Fig. 3A; Fig. S4C through E). Based on this finding, we designated Cgl0579 as ExsI (Esx-secreted iron-binding effector). Ferene S staining further verified its iron-binding ability (Fig. 3B). Site-directed mutagenesis revealed that the Met12 residue was critical for iron-binding, as the ExsI^M12A^ variant exhibited a significantly reduced affinity (*Kd* = 22.18 µM), while substitution of Glu8 (ExsI^E8A^) had no effect (Fig. S4F and G). To examine whether ExsI is a *bona fide* substrate of T7SS, secretion assays were conducted. A plasmid expressing ExsI with a VSV-G epitope tag was introduced into *C. glutamicum* WT, Δ*eccD* mutant, and the *eccD*-complemented strain. Detection of the VSV-G-tagged ExsI in the culture supernatant from the WT but not the Δ*eccD* strain confirmed that ExsI is a secreted protein (Fig. 3C). These results indicate that ExsI is secreted by the T7SS and acts as an iron-binding effector.

Expression of *exsI* was found to be induced under iron-chelated conditions but repressed in iron-rich environments, consistent with its role in iron acquisition (Fig. 3D). Deletion of ExsI significantly reduced intracellular iron levels compared to the WT strain, a phenotype rescued by complementation with functional ExsI but not with the iron-binding-defective ExsI^M12A^ variant (Fig. 3E). Expression of *exsI* was also upregulated in response to H_2_O_2_-induced oxidative stress (Fig. 3F). The Δ*exsI* mutant exhibited increased sensitivity to H_2_O_2_ compared to the WT and complemented strains, and this sensitivity was rescued by the complementation of ExsI but not ExsI^M12A^ (Fig. 3G). Moreover, iron supplementation significantly improved the survival rates of the WT and complemented strains under oxidative stress, while this effect was absent in the Δ*exsI* mutant (Fig. 3H). Exogenous iron reduced ROS levels in the WT and complemented strains, while it had minimal effects on the Δ*exsI* mutant (Fig. 3I). In line with these observations, the Δ*exsI* mutant exhibited more pronounced growth defects under iron-chelated, oxidative stress, or a combination thereof, compared to the WT and complemented strain (Fig. 3J).

Additionally, the Δ*exsI* mutant showed a reduced competitive advantage when co-cultured with *Burkholderia thailandensis* E264 or *Escherichia coli* BL21 under iron-limited conditions (Fig. 3K). This competitive disadvantage was restored by *exsI* complementation (Fig. 3K). Notably, the competitive edge conferred by T7SS-mediated iron acquisition was abolished in iron-rich environments (Fig. 3L). Collectively, these data showed that the T7SS-secreted effector ExsI is an iron-binding protein that is crucial for iron acquisition and confers a competitive edge to *C. glutamicum*.

### ExsI interacts with membrane receptor ExiR to uptake iron

**Fig 4:**
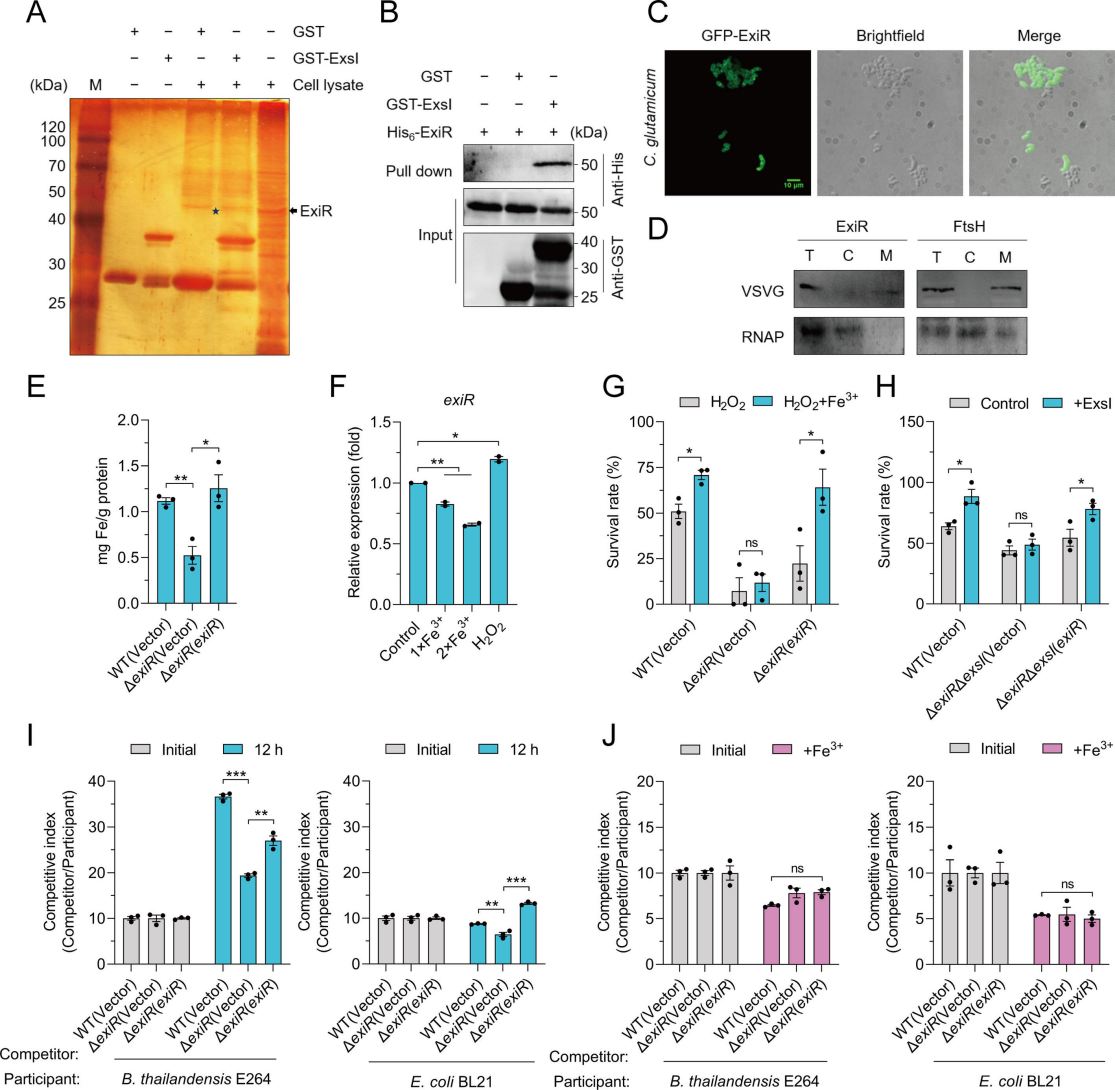
ExsI interacts with membrane receptor ExiR to uptake iron. (**A**) ExiR was retained by agarose beads coated with GST-ExsI. Whole-cell lysate was incubated with GST bind beads coated with GST-ExsI or GST, and PBS buffer was used for washing the uncombined protein. The rest of the protein was detected by SDS-PAGE and silver staining. The bands that were specifically retained by the GST-ExsI-coated beads were identified by mass spectrometry. (**B**) Direct binding of ExsI to ExiR was detected using a GST pull-down assay. GST-ExsI and His_6_-ExiR were co-expressed in *E. coli* BL21 cells. Following cell lysis, the supernatant was captured using GST affinity resin to pull down GST-ExsI. The presence of ExiR proteins and GST-ExsI in the eluate was then detected using an anti-His tag antibody and an anti-GST tag antibody, respectively. *E. coli* BL21 strains co-expressing both His_6_-ExiR and GST, along with strains expressing only His_6_-ExiR, served as negative controls. (**C**) *C. glutamicum* WT strain expressing GFP-ExiR was observed by a high-speed rotary disc-type fluorescence confocal microscope. The scale bar is 10 µm. (**D**) Δ*exiR* mutant expressing ExiR-VSVG was centrifuged at low temperature, samples of each component of the bacteria were collected and separated by SDS-PAGE, and the signals were detected by western blot. FtsH was used as the membrane protein control, and RNAP was used as the cytoplasmic protein control. (**E**) ExiR is involved in the accumulation of intracellular iron under oxidative stress. Relevant late-exponential phase bacterial strains were exposed in an M9 medium containing 15 mM H_2_O_2_ with the addition of Fe^3+^ (1 µM) for 20 min. Iron associated with bacterial cells was measured by atomic absorption spectrometry analysis. (**F**) *C. glutamicum* WT strain was grown in LB medium containing 100 μM Fe^3+^ or 200 μM Fe^3+^ to mid-exponential phase. *C. glutamicum* wild-type cells were grown in an M9 medium containing 15 mM H_2_O_2_ for 5 min. The expression of *exiR* was measured using qRT-PCR. (**G**) Relevant late-exponential phase bacterial strains were cultured in an M9 medium containing 15 mM H_2_O_2_ with or without the addition of Fe^3+^ (1 µM) for 40 min, and the viability of the cells was determined. (**H**) ExiR is required for the resistance to oxidative stress function of ExsI. Relevant late-exponential phase bacterial strains were exposed to 15 mM H_2_O_2_ with the addition of 1 µM Fe^3+^ for 40 min, supplemented with or without 1 µM ExsI protein, and the viability of the cells was determined. (**I**) Bacterial competition between *C. glutamicum* and *B. thailandensis* or *E. coli. C. glutamicum* (competitor) was mixed with *B. thailandensis* (participant) in M9 medium in a ratio of 10:1 and grown at 30°C for 12 h (left). *C. glutamicum* (competitor) was mixed with *E. coli* (participant) in M9 medium in a ratio of 10:1 and grown at 26°C for 12 h (right). Bars represent the mean competitor: participant CFU ratios of three independent experiments (±SEM). (**J**) Bacterial competition between *C. glutamicum* and *B. thailandensis* or *E*. *coli*. *C. glutamicum* (competitor) was mixed with *B. thailandensis* (participant) in M9 medium containing 1 µM Fe^3+^ in a ratio of 10:1 and grown at 30°C for 12 h (left). *C. glutamicum* (competitor) was mixed with *E*. *coli* (participant) in an M9 medium containing 1 µM Fe^3+^ in a ratio of 10:1 and grown at 26°C for 12 h (right). Bars represent the mean competitor: participant CFU ratios of three independent experiments (±SEM). (**A–D**) Representative images from three independent experiments. (**E–J**) Data are mean ± SEM of three independent experiments. *P* values were calculated using a two-tailed Student’s *t*-test for unpaired comparisons or one-way ANOVA with Tukey’s multiple comparisons test. *P < 0.05; **P < 0.01; ***P < 0.001; ns, not significant.

Bacterial-secreted metalloproteins exert their metal ion import ability by interacting with membrane receptors (52, 53). To identify potential receptor proteins for the T7SS-secreted effector ExsI in *C. glutamicum*, we performed a GST pull-down assay by incubating GST-bound beads coated with either GST-ExsI or GST alone with cell lysates of WT *C. glutamicum*. After washing with phosphate-buffered saline (PBS), the retained proteins were analyzed using SDS-PAGE, followed by silver staining. A specific protein of approximately 50 kDa retained by GST-ExsI but not GST alone was identified via mass spectrometry as Cgl0640, designated ExiR (Esx-secreted iron-binding effector receptor) (Fig. 4A). To confirm this interaction, ExiR and ExsI were co-expressed, and a GST pull-down assay validated the interaction between these proteins (Fig. 4B). A bacterial two-hybrid assay further corroborated this interaction (Fig. S5A). Bioinformatic analysis using TMHMM2.0 predicted that ExiR contains three transmembrane domains (Fig. S5B), suggesting its role as a membrane receptor. Confocal microscopy of GFP-tagged ExiR confirmed its localization on the bacterial cell surface (Fig. 4C). Western blot analysis of VSV-G-tagged ExiR in fractionated cell lysates further verified its membrane localization. While ExiR was predominantly detected in the membrane fraction along with the membrane marker FtsH (54), no significant signals were observed in the cytoplasmic fraction containing RNA polymerase as a control (Fig. 4D). These results demonstrate that ExiR is a membrane protein and likely serves as a receptor for ExsI.

To investigate whether ExiR participates in iron acquisition in *C. glutamicum*, the intracellular iron content in an *exiR* deletion mutant (Δ*exiR*) was measured. Deletion of *exiR* significantly reduced intracellular iron levels in *C. glutamicum* compared to the WT strain (Fig. 4E). Expression of *exiR* was repressed under iron-rich conditions but induced by oxidative stress caused by H_2_O_2_ (Fig. 4F). The Δ*exiR* mutant displayed increased sensitivity to H_2_O_2_ compared to the WT and complemented strains, and supplementation of iron significantly improved the survival of WT and complemented strains but not the Δ*exiR* mutant (Fig. 4G). These findings indicate that ExiR facilitates iron uptake and contributes to oxidative stress resistance.

To further investigate the role of ExiR in mediating ExsI-dependent iron uptake, we constructed a double mutant (Δ*exiR*Δ*exsI*) and an *exiR*-complemented strain [Δ*exiR*Δ*exsI*(*exiR*)]. Supplementation of apo-ExsI protein to the culture medium significantly increased the survival of the WT and Δ*exiR*Δ*exsI*(*exiR*) strains but did not affect the Δ*exiR*Δ*exsI* mutant (Fig. 4H). Consistent with this observation, intracellular iron levels showed a similar increase pattern in these strains (Fig. S6A). These results suggest that ExiR is required for the function of ExsI in facilitating iron transport. We further examined the growth of WT *C. glutamicum*, the Δ*exiR* mutant, and the complemented Δ*exiR*(*exiR*) strain under iron-chelated and oxidative stress conditions. Under normal growth conditions, all strains showed similar growth (Fig. S6B). However, under iron-chelated conditions induced by EDDHA, the Δ*exiR* mutant exhibited significantly reduced growth compared to the WT and complemented strains (Fig. S6C). This defect was further exacerbated under combined iron-chelated and oxidative stress conditions (H_2_O_2_ plus EDDHA) (Fig. S6D). Bacterial competition assays revealed that the Δ*exiR* mutant had a significantly reduced competitive advantage against *Burkholderia thailandensis* E264 and *Escherichia coli* BL21 under iron-limited conditions (Fig. 4I). The addition of iron abolished the competitive advantage of the WT strain, confirming the importance of ExiR-mediated iron uptake in bacterial competition (Fig. 4J). The BLASTP analysis revealed that the ExiR homologs are widely distributed in Actinobacteria (Fig. S7A and B), suggesting that the ExiR-mediated iron uptake mechanism may be widespread. These results indicate that ExiR is a membrane-localized receptor protein that interacts with the T7SS-secreted effector ExsI to facilitate iron uptake.

### ExsI homologs in diverse bacteria contribute to resisting oxidative stress by importing iron

**Fig 5:**
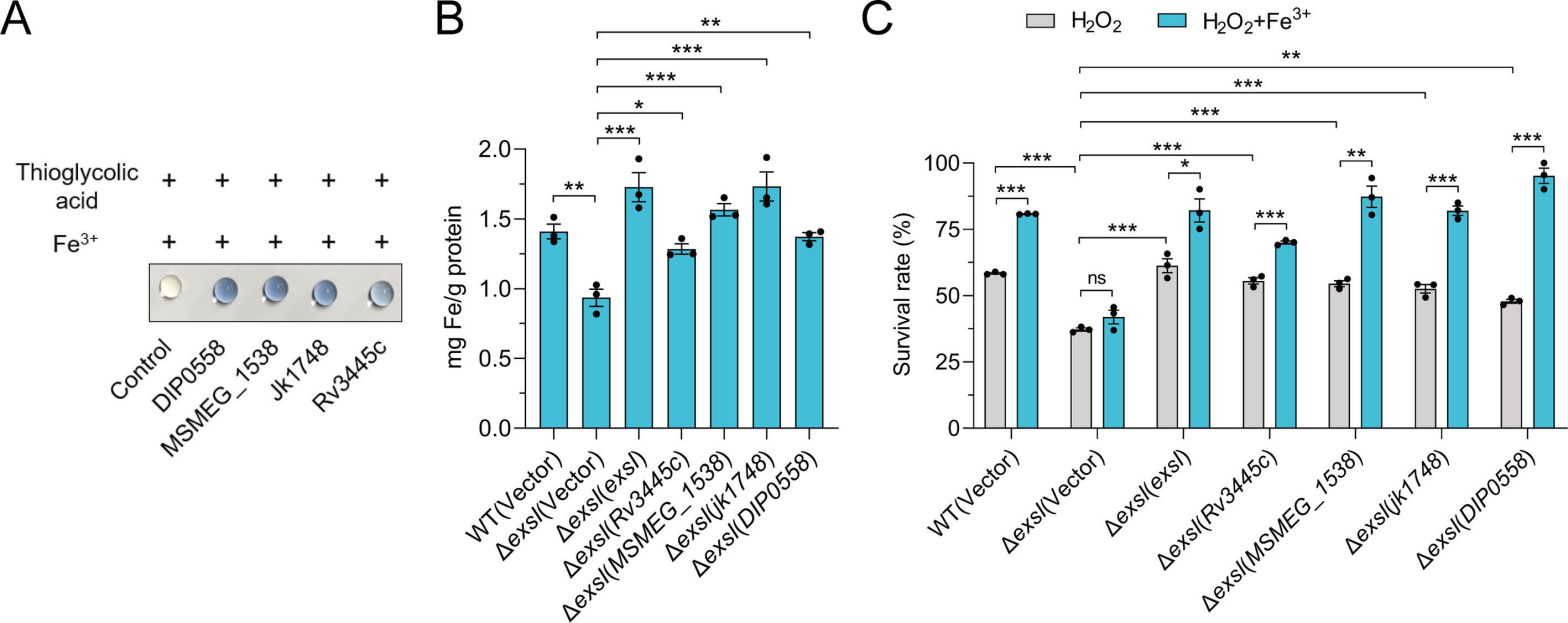
ExsI homologs in diverse bacteria contribute to resisting oxidative stress by importing Fe^3+^. (**A**) The ability of ExsI homologs to bind Fe^3+^ was assessed using a Ferene S staining assay. The proteins were mixed with the stain, dot-blotted onto a nitrocellulose membrane, and then observed for the development of a blue color. Representative images from three independent experiments. (**B**) ExsI homologs are involved in the accumulation of intracellular iron under oxidative stress in *C. glutamicum*. Relevant late-exponential phase bacterial strains were exposed in an M9 medium containing 15 mM H_2_O_2_ with the addition of 1 μM Fe^3+^ for 20 min. Iron associated with bacterial cells was measured by atomic absorption spectrometry analysis. (**C**) ExsI homologs are involved in the alleviation of the sensitivity of the Δ*exsI* mutant to H_2_O_2_ by the addition of exogenous Fe^3+^. Relevant late-exponential phase bacterial strains were cultured in an M9 medium containing 15 mM H_2_O_2_ with or without the addition of 1 µM Fe^3+^ for 40 min, and the viability of the cells was determined. (**B–C**) Data are mean ± SEM of three independent experiments. *P* values were calculated using a two-tailed Student’s *t*-test for paired comparisons or one-way ANOVA with Tukey’s multiple comparisons test. *P < 0.05; **P < 0.01; ***P < 0.001; ns, not significant.

To determine the evolutionary conservation of ExsI among diverse bacterial species, we retrieved 1,047 homologous protein sequences from the NCBI non-redundant protein database. Phylogenetic analysis, based on one representative sequence per order, revealed that over 95% of ExsI homologs are distributed within the class Actinobacteria, with a strong enrichment in the order *Mycobacteriales* (Fig. S8A and B). This finding underscores the high conservation of ExsI within Actinobacteria and its potential physiological importance. Further comparative analysis of T7SS gene clusters from representative pathogenic and non-pathogenic species (*Mycobacterium tuberculosis* H37Rv, *Mycolicibacterium smegmatis* mc^2^155, *Corynebacterium jeikeium*, and *Corynebacterium diphtheriae* NCTC 13129) revealed that the structure and organization of the T7SS loci are highly similar to those of *C. glutamicum* (Fig. S8C). Multiple sequence alignment of ExsI homologs confirmed the presence of the conserved WXG motif and a critical iron-binding residue at position 12 (Met12) (Fig. S8D). To evaluate the functional conservation of ExsI homologs, we performed a Ferene S staining assay to test the iron binding capacity of ExsI proteins from *M. tuberculosis* (Rv3445c), *M. smegmatis* (MSMEG_1538), *C. jeikeium* (Jk1748), and *C. diphtheriae* (DIP0558). The results demonstrated that all homologs effectively bind iron, indicating a conserved mechanism for T7SS-mediated iron uptake across diverse bacterial species (Fig. 5A).

To assess the function of ExsI homologs, we heterologously expressed these proteins in the *C. glutamicum* Δ*exsI* mutant and measured intracellular iron content. Expression of ExsI homologs from *M. smegmatis*, *C. jeikeium*, and *C. diphtheriae* significantly restored intracellular iron levels in the Δ*exsI* mutant, though to varying extents (Fig. 5B). These results suggest that ExsI homologs from diverse bacteria can substitute for *C. glutamicum* ExsI in facilitating iron uptake. To investigate the role of ExsI homologs in oxidative stress resistance, we tested the survival of *C. glutamicum* Δ*exsI* strains expressing homologous proteins under H_2_O_2_-induced oxidative stress. Expression of ExsI homologs significantly increased the survival rates of the Δ*exsI* mutant under oxidative stress conditions (Fig. 5C). The addition of exogenous iron further enhanced the survival rates of WT and Δ*exsI* strains expressing different ExsI homologs. However, the Δ*exsI* mutant lacking any homolog exhibited significantly lower survival, highlighting the role of ExsI homologs in oxidative stress adaptation (Fig. 5C). Together, these data indicated that ExsI homologs are widespread and could enhance iron accumulation and contribute to oxidative stress resistance.

### ExsI homolog in *M. smegmatis* contributes to the virulence of bacteria *in vitro* and *in vivo*

**Fig 6:**
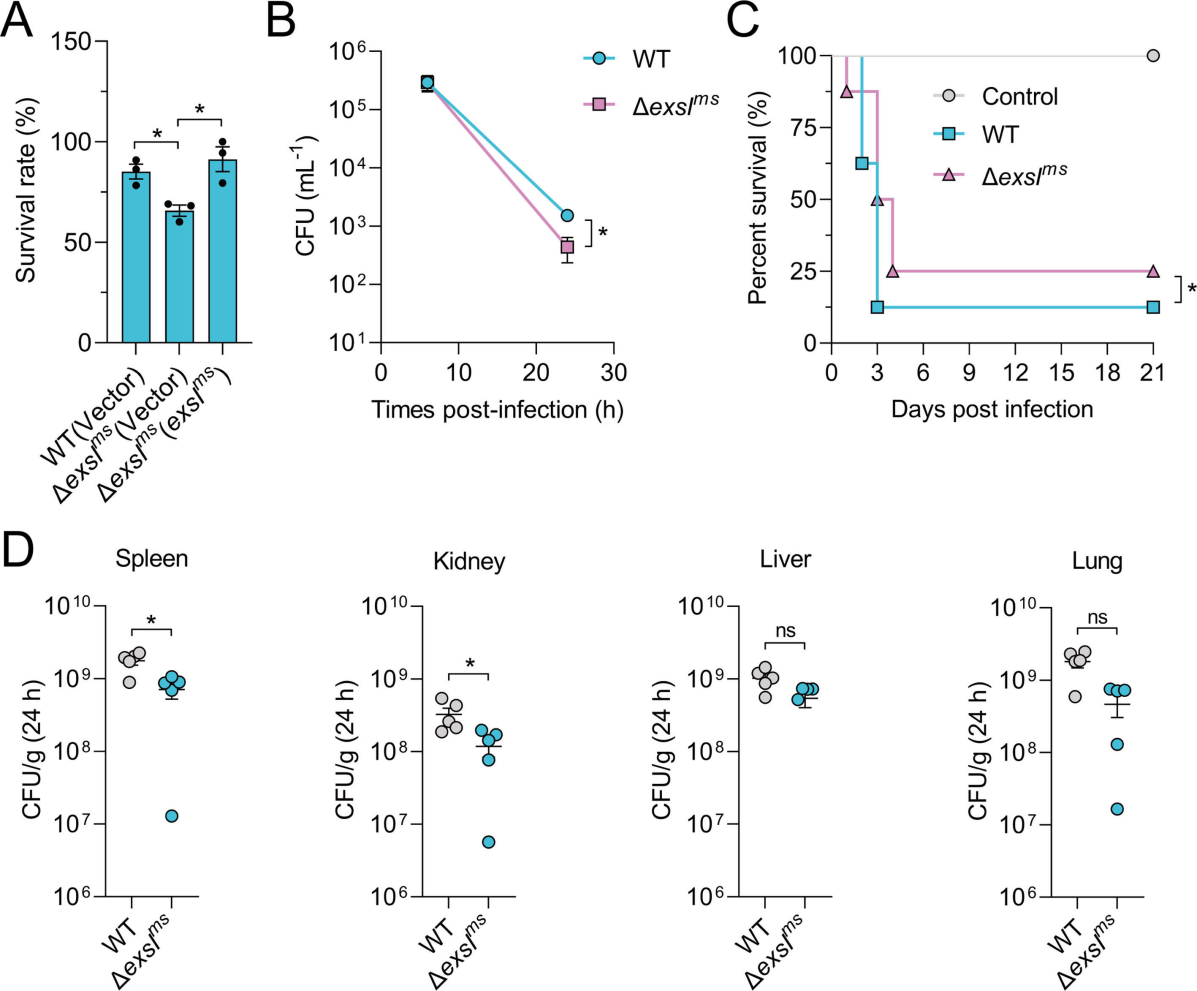
ExsI homolog in *M. smegmatis* contributes to the virulence of bacteria *in vitro* and *in vivo*. (**A**) Relevant late-exponential phase bacterial strains were cultured in M9 medium containing 5 mM H_2_O_2_ for 6 h, and the viability of the cells was determined. Data are mean ± SEM of three independent experiments. (**B**) RAW264.7 cells were infected with WT *M. smegmatis* and Δ*exsI^ms^* at a multiplicity of infection of 10:1; after 6 or 24 h infection, the CFUs of intracellular *M. smegmatis* cells were determined by plating appropriate dilutions on 7H10 agar plates. Data are mean ± SEM of three independent experiments. (**C**) C57BL/6 mice were infected intraperitoneally with WT *M. smegmatis* and Δ*exsI^ms^*, and survival was monitored daily (*n* = 8). Data were illustrated as a percentage of mice survival. (**D**) C57BL/6 mice were infected intraperitoneally with 3 × 10^9^ CFU of WT *M. smegmatis* and Δ*exsI^ms^*. C57BL/6 mice were sacrificed after 24 h post-infection, and the bacterial burdens in livers, spleens, kidneys, and lungs were counted (*n* = 5). *P* values in (**A–B**) were calculated using a two-tailed Student’s *t*-test for paired comparisons or one-way ANOVA with Tukey’s multiple comparisons test. *P* values in (**C**) were analyzed using the log-rank (Mantel-Cox) test. *P* values in (**D**) were analyzed using the Mann-Whitney test. Error bars represent ±SEM. *P < 0.05; ns, not significant.

*M. smegmatis* mc^2^155 contains three T7SS operons, ESX-1, ESX-3, and ESX-4, with the *exsI* homolog gene *MSMEG_1538* (*exsI^ms^*) located in the ESX-4 locus. To evaluate the role of ExsI^ms^ (*MSMEG_1538*, an ExsI homolog) in oxidative stress resistance, we measured the survival rates of the Δ*exsI^ms^* mutant and WT *M. smegmatis* under oxidative stress conditions. The Δ*exsI^ms^* mutant exhibited reduced survival compared to the WT strain, while both strains showed similar growth rates in iron-rich 7H9 medium (Fig. 6A; Fig. S9). Next, the role of *exsI^ms^* in bacterial pathogenicity was assessed using an *in vitro* infection model with RAW264.7 macrophage cells. At 6 h post-infection, intracellular bacterial load for both WT and Δ*exsI^ms^* strains was similar, indicating successful infection of macrophages (Fig. 6B). However, at 24 h post-infection, the survival rate of the Δ*exsI^ms^* mutant within macrophages was significantly lower than that of the WT strain, demonstrating that ExsI^ms^ enhances the ability of *M. smegmatis* to persist within macrophages.

Furthermore, mice were intraperitoneally injected with 3 × 10^9^ colony-forming units (CFU) of either the WT *M. smegmatis* or the Δ*exsI^ms^* mutant, and mortality was monitored over 21 days. Mice infected with the WT strain exhibited higher mortality rates compared to those infected with the Δ*exsI^ms^* mutant (Fig. 6C). Additionally, bacterial CFU in the spleen and kidneys of mice infected with the WT strain were significantly higher than those in mice infected with the Δ*exsI^ms^* mutant at 24 h post-infection, suggesting that the absence of ExsI^ms^ reduces the ability of *M. smegmatis* to infect (Fig. 6D). These results demonstrate that ExsI^ms^ enhances the virulence of *M. smegmatis in vivo*.

### ExsI homolog in *M. smegmatis* facilitates the bacteria to counteract host nutritional immunity

**Fig 7:**
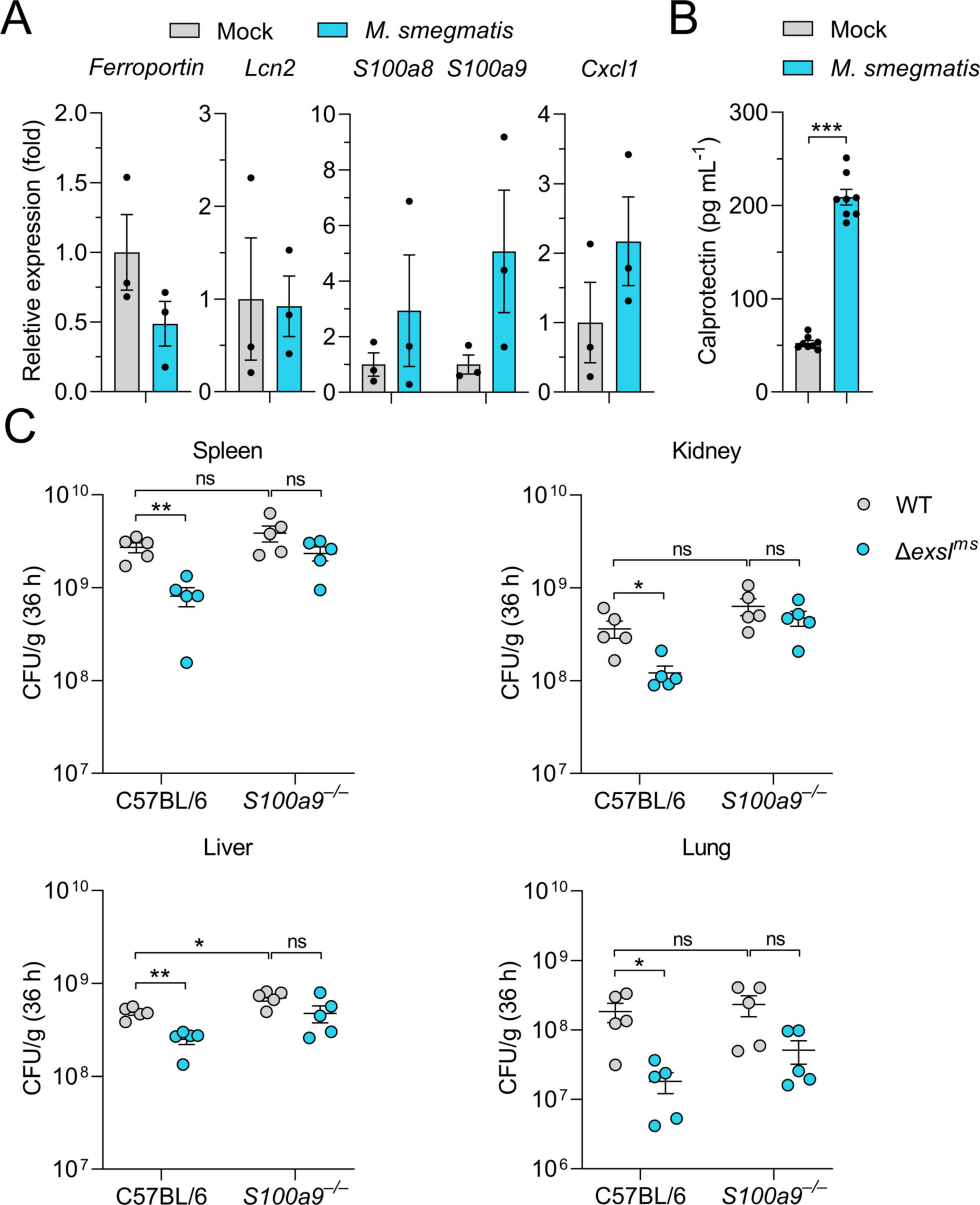
ExsI homolog in *M. smegmatis* facilitates the bacteria to counteract host nutritional immunity. (**A**) The mRNA expression level of *Ferroportin*, *Lcn2*, *S100a8*, *S100a9,* and *Cxcl1* in the spleen samples of mice 36 h post-infection with wild-type *M. smegmatis* was determined by qRT-PCR. Data are expressed as fold decrease/increase over mock-infected WT mice. (**B**) The concentration of calprotectin detected by enzyme-linked immunosorbent assay (ELISA) in mice 36 h post-infection with *M. smegmatis* or mock control is shown (*n* = 8). (**C**) C57BL/6 and *S100a9^−/−^* mice were separately infected intraperitoneally with 3 × 10^9^ CFU of either WT *M. smegmatis* or Δ*exsI^ms^*. After 36 h post-infection, the spleens, kidneys, livers, and lungs were harvested under sterile conditions. The organs were homogenized and plated on 7H10 agar plates with appropriate dilution (*n* = 5). *P* values in (**B**) were calculated using a two-tailed Student’s *t*-test for paired comparisons. *P* values in (**C**) were analyzed using the Mann-Whitney test. Error bars represent ±SEM. *P < 0.05; **P < 0.01; ***P < 0.001; ns, not significant.

Intracellular iron homeostasis in the host is regulated by Ferroportin (55). We observed a trend of *ferroportin* downregulation in the spleens of mice infected with *M. smegmatis* (Fig. 7A), which may suggest a potential alteration in the intracellular iron pool in the infected cells. Lipocalin-2 (LCN2) is a prominent antimicrobial protein released during bacterial infections that limits bacterial access to iron by sequestering siderophores (56, 57). However, we did not observe clear induction of *Lcn2* in the spleens of *M. smegmatis*-infected mice. We also found that expression of *S100a8* and *S100a9* appeared elevated in the spleens of infected mice (Fig. 7A). This may indicate a potential increased inflammatory response, as evidenced by elevated *Cxcl1* transcript levels. Consistently, the Calprotectin in *M. smegmatis*-infected mice serum was also significantly increased (Fig. 7B).

The role of ExsI^ms^ in counteracting CP-mediated nutritional immunity was further assessed by infecting calprotectin-deficient (*S100a9^–/–^*) and WT C57BL/6 mice with either WT or Δ*exsI^ms^* strains. In C57BL/6 mice, the WT strain exhibited significantly higher bacterial counts in the spleen, kidneys, liver, and lungs compared to the Δ*exsI^ms^* mutant (Fig. 7C). In *S100a9^–/–^* mice, both WT and mutant strains showed enhanced infection compared to infections in C57BL/6 mice, reflecting the role of nutritional immunity in defending against bacterial infection. Importantly, the difference in bacterial infection between the WT and mutant strains observed in C57BL/6 mice was dwarfed in *S100a9^–/–^* mice, underscoring the role of ExsI^ms^ in overcoming CP-mediated iron sequestration (Fig. 7C). This suggests that ExsI^ms^ plays an important role in helping *M. smegmatis* to counteract host nutritional immunity.

## DISCUSSION

T7SS is a secretion mechanism that allows Gram-positive bacteria to thrive under intense pressures, whether they originate from competing microbes or the host immune system (5). While T7SSs are known to secrete distinct substrates, much of the emphasis has been on the delivery of virulence factors or immunomodulatory proteins (5, 58), rather than on secreted effectors specialized in scavenging key micronutrients from the extracellular milieu. Our study reveals that T7SS can drive iron uptake through the secretion and recapture of a dedicated iron-scavenging effector, ExsI, providing *C. glutamicum* with a strategy for securing critical nutrients under challenging conditions. By secreting ExsI to uptake iron, T7SS plays essential roles in nutrient acquisition, oxidative stress resistance, bacterial exploitative competition, and evasion of host nutritional immunity. These findings reshape our understanding of T7SS as a multifaceted secretion apparatus by secreting and re-importing an iron scavenger.

While T7SS has been implicated in bacterial nutrient acquisition through pore-forming or membrane-integrated transport proteins (20, 32, 34), our work reveals a distinct mechanism in *C. glutamicum*. We demonstrated that the T7SS secretes ExsI, a WXG100 family effector that functions as a secreted high-affinity iron scavenger. The ExsI-iron complex is subsequently recognized and engaged by the membrane-localized receptor ExiR, thereby completing the extracellular steps of a putative iron acquisition pathway. This extracellular iron-ferrying system, activated under iron-limited conditions in a Fur-dependent manner (Fig. 1), actively captures and delivers essential metal ions into bacterial cells via surface receptors, contrasting the traditional siderophore secretion-recapture paradigm of iron uptake in Gram-positive bacteria (59). The ExsI-ExiR system offers multiple evolutionary benefits. By circumventing siderophore production, this system avoids the risk of nutrient piracy characteristic of siderophore-mediated iron acquisition (60, 61). The presence of a dedicated receptor not only enables efficient iron retrieval but also prevents resource exploitation by competing bacteria (62). Additionally, this mechanism potentially accounts for the rapid utilization of iron from YukE-induced root leakage (36). The ExsI-ExiR system not only advances our understanding of T7SS functionality by demonstrating effector-driven nutrient acquisition but also provides new insights into bacterial iron homeostasis and ecological adaptation. This discovery expands the known functional repertoire of T7SS beyond classical pore-forming or membrane-integrated transport proteins, revealing a sophisticated effector-driven strategy for resource acquisition in competitive environments.

Intense competition, including interference and exploitative competition, can be found in all bacterial communities. Interference competition refers to direct antagonistic interactions, where one organism delivers harmful substances like toxins to competitors, thereby reducing their viability. Exploitative competition, in contrast, occurs indirectly through the depletion of shared resources, allowing one organism to outcompete others by securing a greater share of these resources. T7SS, especially in mycobacteria, has been described for its role in interference competition interactions through the translocation of toxic effectors directly into competitor cells (63–65). Our data reveal an unappreciated T7SS-mediated exploitative competition mechanism, which is based on the more efficient uptake or utilization of limited resources in the environment (62). We reported the first example of T7SS conferring a nutrient-based exploitative advantage. Instead of attacking rival bacteria directly, *C. glutamicum* leverages its T7SS-secreted ExsI to secure iron from the environment, reinforcing that resource preemption is a critical, previously underappreciated function of T7SS. This monopolization of resources ensures that *C. glutamicum* secures vital micronutrients from its competitors, thereby gaining a growth advantage and outcompeting other bacteria. The finding of exploitative competition mediated by T7SS prompted us to speculate that, beyond toxicity-based antagonism, T7SS can also modulate resource availability. The ability of T7SS to secrete a diffusible iron-binding effector allows a bacterium to “mine” the extracellular environment for a scarce resource. Such a strategy can be especially powerful in nutrient-limited niches, especially in environmental habitats or host tissues. This conceptual shift encourages a reexamination of the roles of T7SS effectors in other organisms, as T7SS orthologs and associated WXG100 proteins could similarly function to scavenge diverse ions, thereby shaping competitive outcomes in complex microbial consortia. Supportively, it has been reported that T6SS-mediated competition profoundly changes the gut microbiota of the host (62). However, the role of T7SS in altering community composition and potentially tipping the competitive balance in mixed microbial environments needs further investigation.

Oxidative stress resistance is a fundamental trait that dictates bacterial survival in hostile environmental conditions or within host cells. Previous research has established T6SS as a key player in mitigating oxidative stress (52, 66, 67). However, there has been no direct evidence that T7SS contributes to redox homeostasis. Our results strongly link T7SS-dependent iron acquisition to the ability of *C. glutamicum* to withstand oxidative challenges. We showed that the T7SS-deficient mutants exhibited impaired growth under conditions that combined oxidative stress with low iron availability (Fig. 2). Further investigation revealed that the ExsI-ExiR-mediated iron uptake is also involved in oxidative stress resistance (Fig. 3). The ability to handle oxidative stress through improved iron uptake suggests that T7SS is not merely a specialized system for pathogenic or antagonistic interactions; it also supports fundamental cellular survival strategies in hostile environments. By linking T7SS to the oxidative stress response through a nutrient acquisition strategy, our study introduces a crucial new facet of T7SS physiology. Bacteria often face oxidative challenges from competing microorganisms or hostile host environments. Hence, T7SS emerges as an integral system that aligns nutrient acquisition strategies with defense mechanisms.

Iron plays a dual role in oxidative stress responses, serving as both an essential cofactor for antioxidant enzymes, such as catalase and superoxide dismutase, that confer cellular protection and a catalyst for ROS generation through the Fenton reaction that exacerbates oxidative damage (68, 69). Our findings demonstrate that the ExsI-ExiR-mediated iron uptake enhances oxidative stress resistance in *C. glutamicum* (Fig. 3H and 4G), an observation that may appear counterintuitive given iron’s capacity to worsen oxidative stress. This apparent paradox can be explained by the concentration-dependent effects of iron. As observed in *Ralstonia eutropha* JMP134, low iron concentrations (1–10 μM) improve oxidative stress resistance through enhanced catalase activity, while higher concentrations (20 µM) abolish this protection (70). Consistent with this model, exogenous iron reduced ROS levels in wild-type and complemented strains but not in the Δ*eccC* and Δ*exsI* mutants (Fig. 2B and 3I), suggesting that the T7SS and ExsI confer protection likely through controlled iron acquisition that modulates the activities of iron-dependent antioxidant enzymes, thereby avoiding the detrimental effects of iron overload.

CP is a well-known player in the nutritional tug-of-war between host and pathogens by reducing the availability of divalent cations (Zn^2+^, Mn^2+^), and often iron (Fe^3+^) availability (39, 40). This study provides the first evidence that T7SS can facilitate bacterial subversion of CP-mediated nutritional immunity. The T7SS-mediated iron acquisition contributes to bacterial fitness and virulence, as *M. smegmatis* displayed reduced survival in macrophages and attenuated virulence in mice when its ExsI homolog was deleted. Remarkably, the advantage conferred by T7SS-mediated iron acquisition in a CP-deficient host was substantially diminished (Fig. 7). This demonstrates that T7SS not only scavenges iron from the extracellular environment but also can counteract host-imposed iron withholding. Differing from the previously described T7SS-mediated capabilities of circumventing host innate immunity (5), this study uncovered an unappreciated role of T7SS in overcoming CP-mediated nutritional immunity. This represents an important addition to the T7SS toolbox, highlighting its versatility not just in antagonistic interactions or virulence factor delivery but also in enabling bacterial survival under host-imposed nutrient deprivation.

Given that ancestral ESX-4 loci are conserved in mycobacteria and related taxa (1, 43), and phylogenetic analyses indicate that homologs of effector (ExsI) and receptor (ExiR) are broadly distributed and highly conserved (Fig. 5), it is plausible that pathogenic bacteria exploit T7SS in the same way. This would align with the fundamental importance of ESX-4 in bacterial physiology and could partially explain why ESX-4 is so widely maintained despite intense host immune pressure (5). Securing essential minerals like iron and mitigating oxidative damage would provide a strong selective benefit. Considering that both pathogenic species (e.g., *M. tuberculosis* and *M. smegmatis*) and environmental organisms (*C. glutamicum*) face challenges, such as limited trace metals and oxidative stress in their respective niches, our findings imply that T7SS may serve as a key system for coping with diverse environmental pressures. More importantly, the evolutionary parallel with T6SS’s established roles in metal acquisition and oxidative stress resistance suggests that these distinct secretion systems have evolved similar mechanisms to secure vital nutrients while fending off hostile conditions.

Although our study demonstrates that the T7SS-secreted effector ExsI binds Fe^3+^ extracellularly and interacts with the membrane receptor ExiR to enable iron uptake, the precise mechanism of iron translocation across the inner membrane remains unresolved. ExiR, predicted to contain three transmembrane domains, likely serves as a receptor, but it is unclear whether it functions autonomously as a transporter or integrates into a multi-subunit complex akin to ABC-type permeases in other iron acquisition systems. Potential accessory factors, such as chaperones for iron release, ATP-binding cassettes for energization, or additional membrane proteins, have not been identified, limiting our understanding of the pathway’s energetics and specificity. Future work should focus on experimentally delineating these components, in particular through cryo-EM structural analysis of the ExsI-ExiR complex and proteomic screens for interactors. Addressing these gaps will clarify how T7SS orchestrates active iron import and its broader implications for bacterial metal homeostasis.

While our findings achieve statistical significance (*P* < 0.05), several assays, including bacterial sensitivity to oxidative stress, intracellular ROS quantification, and intracellular iron content measurements, displayed modest effect sizes relative to wild-type controls. This limitation could arise from (i) intrinsic biological features of *C. glutamicum*, such as its stringent control over iron homeostasis and oxidative stress pathways, which may attenuate phenotypic differences, or (ii) methodological constraints, including the fleeting dynamics of ROS or the detection limits of standard techniques like atomic absorption spectrometry. For example, although our approaches yielded consistent and reproducible data, implementing higher-resolution methods, such as inductively coupled plasma mass spectrometry, in subsequent research could better capture subtle iron variations. Nonetheless, the uniform direction and statistical robustness of these effects across independent replicates affirm their biological validity, with the modest scale potentially mirroring the precise regulatory equilibrium in *C. glutamicum*’s iron metabolism and stress response networks.

In sum, our study reveals that T7SS is not merely a conduit for protein export or a specialized virulence machine. Instead, it emerges as an active iron uptake system that underwrites bacterial survival and ecological success in multiple ways, encompassing oxidative stress protection, exploitative nutrient competition, and resistance to host nutritional immunity. These findings transform our understanding of T7SS from a niche secretion apparatus into a multifaceted tool that integrates versatile functions.

## MATERIALS AND METHODS

### Bacterial strains and growth conditions

*Corynebacterium glutamicum* ATCC13032 was cultured in Luria Bertani (LB) (Sangon Biotech, Shanghai, China) or M9 medium (Na_2_HPO_4_, 6 g L^–1^; KH_2_PO_4_, 3 g L^–1^; NaCl, 0.5 g L^–1^; NH_4_Cl, 1 g L^–1^; MgSO_4_, 1 mM; CaCl_2_, 0.1 mM; Glucose 0.2%) aerobically on a rotary shaker (220 rpm) at 30°C. *Burkholderia thailandensis* E264 and *Escherichia coli* BL21 were cultured in LB broth or M9 medium aerobically on a rotary shaker (220 rpm) at 37°C or 30°C, respectively. Brain Heart Infusion broth (Solarbio, Beijing, China) was used for culturing *C. glutamicum* after electroporation. *M. smegmatis* was grown in 7H9 broth (hopebio, Tsingtao, China) supplemented with 0.05% Tween 80, 0.2% glycerol, and 10% ADC (hopebio) or on 7H10 agar (hopebio) supplemented with 0.05% Tween 80, 0.2% glycerol, and 10% OADC (hopebio). Anhydrotetracycline (100 ng mL^–1^) was added to the *M. smegmatis* cultures when needed. Appropriate antibiotics were included in the growth medium, and their corresponding concentrations are Ampicillin (100 µg mL^–1^), Kanamycin (50 µg mL^–1^), Chloramphenicol (20 µg mL^–1^), Nalidixic acid (20 µg mL^–1^), and Hygromycin (50 µg mL^–1^ for *M. smegmatis*, 100 µg mL^–1^ for *E. coli*). All bacterial strains are listed in Table S1 (see Text S2 Supplementary Tables file).

### Mice

Mice on a C57BL/6 background were procured from Beijing Vital River Laboratory Animal Technology Co., Ltd., from China. All mice-related procedures adhered to the Regulations for the Administration of Affairs Concerning Experimental Animals, approved by the State Council of the People’s Republic of China. The study protocol received approval from the Animal Welfare and Research Ethics Committee of Northwest A&F University (Protocol number: XN2023-1004). The mice were maintained in a controlled environment with a temperature of 24°C ± 2°C, humidity of 50% ± 10%, air exchange of 35 cycles, and a 12 h light-dark cycle. They had *ad libitum* access to food and water and were housed in specific pathogen-free conditions. *S100a9*^–/–^ mice were purchased from Cyagen Inc., China (https://www.cyagen.com/). To evaluate the bacterial load in various organs, mice were sacrificed 72 h post-infection. Tissues were weighed and homogenized in 0.9% NaCl, and serial dilutions of the homogenates were plated on Arga plates.

### Mice infection

Six-week-old female C57BL/6 or *S100a9*^–/–^ mice were housed under 12/12 h light/dark cycles with pathogen-free food and water. After 7 days of acclimatization, the mice were randomly divided into two groups and injected intraperitoneally with 3 × 10^9^ CFUs of *M. smegmatis* wild type and Δ*exsI^ms^*, respectively. The cells were plated on 7H10 agar plates to determine the CFU before administration. At 24/36 h post-infection, mice were dissected, and the spleens, livers, kidneys, and lungs were harvested under sterile conditions. Organs were weighed and homogenized in 0.9% sodium chloride injection containing 0.05% Tween 20, and homogenates were diluted appropriately and plated onto 7H10 agar plates. The colonies were counted after the cells had been cultured for 3−5 days in a 37°C incubator. 6-week-old female C57BL/6 mice were infected intraperitoneally with 3 × 10^9^ CFUs *M. smegmatis* wild type and Δ*exsI^ms^*. Infected mice were observed three times daily for survival, and survival rates were calculated for each group. As the control, mice were also injected intraperitoneally with 0.9% sodium chloride injection to observe the survival rate.

### Cell lines

Raw264.7 cells used in this study were cultured in DMEM (high-glucose), supplemented with 10% fetal bovine serum (Beyotime), penicillin (100 U mL^–1^), and streptomycin (100 µg mL^–1^).

### Bacterial two-hybrid assay

Bacterial two-hybrid assays were carried out as previously described (71, 72). Briefly, pUT18C-*exsI* and pKT25-*exiR* were co-transformed into *E. coli* BTH101 competent cells. Meanwhile, pUT18C/pKT25, pUT18C-*exsI*/pKT25, pUT18C/pKT25-*exiR,* or pUT18C-zip/pKT25-zip were co-transformed into *E. coli* BTH101 competent cells as negative and positive controls, respectively. The transformants were plated on MacConkey agar plates supplemented with ampicillin (100 µg mL^–1^) and kanamycin (50 µg mL^–1^) and 1 mM IPTG. After three successive generations, the red colonies indicate an interaction, and the white colonies indicate no interaction. Efficiencies of interaction between different proteins were quantified by measuring β-galactosidase activities in liquid cultures. Briefly, overnight cultures were diluted 100-fold into fresh 3 mL LB broth containing 1 mM IPTG and antibiotics at 30°C until the last exponential phase, and β-galactosidase activities were assessed using O-nitrophenyl-β-galactoside as the substrate.

### Protein secretion assay

Protein secretion assay for ExsI was performed according to the described methods with minor modifications (73, 74). Briefly, *C. glutamicum* strains expressing ExsI-VSVG were cultured in 3 mL LB broth at 30°C overnight to reach the stationary phase and diluted 100-fold into fresh 500 mL LB broth containing 1 mM IPTG and appropriate antibiotics until the late-exponential phase. Collected 1 mL of cells served as the whole-cell lysate sample and were resuspended in 100 µL SDS loading buffer. The remaining cultures were centrifuged at 4,500 rpm for 15 min, followed by centrifugation of the supernatant at 6,000 rpm for 15 min, and 8,000 rpm for 30 min. To remove residual bacterial cells, the culture supernatant was filtered with a 0.22 µm pore size filter (Millipore, MA, USA). All the secreted proteins in the culture supernatant were enriched three times through a nitrocellulose filter (BA85, Whatman, Germany). The nitrocellulose filter was cut into pieces and soaked in 100 µL SDS loading buffer with heat treatment at 65°C for 15 min and then boiled for 15 min to recover the proteins present. All protein samples were separated by SDS-PAGE and detected by western blot analysis. All samples were normalized to the OD_600_ of the culture and volume used in the preparation.

### Metal-free ExsI protein preparation

Metal-free ExsI protein preparation was performed as described previously with minor modifications (53). Briefly, the purified ExsI protein (100 µM) was added to the solution containing 25 mM Tris, 25 mM diethylene triamine pentaacetic acid, and 10% glycerol at pH 7.5 to remove as many ions as possible. After 1 h, the protein was dialyzed with the solution (25 mM Tris, 10% glycerol, pH 7.5) at 4°C overnight. The resulting protein was stored at –80°C until use.

### Isothermal titration calorimetry

Metal ion-binding was measured using ITC as previously described with minor modifications (75). To confirm the Fe^3+^-binding ability of ExsI, the metal-free ExsI protein was measured with a NANO-ITC 2G microcalorimeter (TA Instruments, USA) at 25°C, according to the manufacturer’s instructions. Titrations were carried out with Fe^3+^ in the syringe and ExsI in the cell. Each titration experiment consisted of 25 injections with 300 s intervals between each injection. Data reduction and analysis were performed with the Nano Analyze software (TA Instruments), fitting them to an independent binding model.

### Quantitative real-time PCR

Quantitative real-time PCR analysis was performed as described previously with minor modifications (76). Briefly, *C. glutamicum* strains were cultured in 3 mL LB broth at 30°C overnight to reach the stationary phase and diluted 100-fold into fresh 3 mL LB broth containing 1 mM IPTG and different reagents (H_2_O_2_, Fe^3+^, EDDHA) until mid-exponential phase. A total of 500 µL cells were harvested, and their total RNA was extracted using the RNAprep Pure Cell/Bacteria Kit (TIANGEN, Beijing, China) along with the DNase I Kit (Sigma-Aldrich, Taufkirchen, Germany). Mice tissue RNA isolation utilized the RNAprep Pure Tissue Kit (TIANGEN, #DP431). Quantitative reverse transcription PCR (qRT-PCR) was used to measure the expression of different genes (*eccB*, *mycP*, *eccD*, *eccC*, *exsI*, *exiR, Ferroportin*, *Lcn2*, *S100a8*, *S100a9*, *Cxcl1*). The first-strand cDNA was obtained using the EasyScript One-Step gDNA Removal and cDNA Synthesis SuperMix (TransGen Biotech, Beijing, China). Reverse transcription was performed at 25°C for 10 min, followed by 42°C for 15 min and 85°C for 5 s. qRT-PCR was performed on the LightCycler 96 System (Roche) with *PerfectStart* Green qPCR SuperMix (TransGen Biotech). The primers used in qRT-PCR are listed in Table S2 (see Text S2 Supplementary Tables file). 16S rRNA was used as an internal standard to normalize relative abundance in bacterial cells (77). The housekeeping gene GAPDH was used as the internal standard for mammalian cells.

### GST pull-down assay

The GST pull-down assay was carried out as previously described with minor modifications (67). Briefly, to find proteins interacting with ExsI, GST bind beads coated with 0.1 mg GST-ExsI were incubated with whole cell lysate on a rotator overnight at 4°C, and GST protein was performed with the same procedures as a negative control. The beads were harvested and washed three times with PBS buffer containing 300 mM NaCl and then washed three times with PBS buffer containing 500 mM NaCl. The beads were resuspended in 100 µL SDS loading buffer with heat treatment at 100°C for 15 min. The samples were detected by SDS-PAGE and silver stain (Bio-Rad). The different bands between GST-ExsI and GST protein were digested with trypsin and analyzed by matrix-assisted laser desorption/ionization/mass spectrometry (Voyager-DE STR, Applied Biosystems, Waltham, MA), and the resulting data are listed in Supplementary Data 1.

To test the interactions between ExiR and ExsI, plasmids pET28a-*exiR* and pGEX-6p-1-*exsI* were co-expressed in *E. coli* BL21 (DE3) cells. The resulting strain was cultured in 500 mL LB broth containing 0.25 mM IPTG at 22°C overnight. Following cell lysis, the supernatant was captured using GST affinity resin to pull down GST-ExsI. The presence of His_6_-ExiR proteins and GST-ExsI in the eluate was then detected using an anti-His tag antibody and an anti-GST tag antibody, respectively. Meanwhile, pET28a-*exiR* or pET28a-*exiR*/pGEX-6p-1 was expressed in *E. coli* BL21 (DE3) cells as negative controls, respectively.

### Western blot analysis

The western blot analysis was carried out as previously described with minor modifications (52). Briefly, samples were separated by SDS-PAGE and transferred onto polyvinylidene fluoride membranes (Millipore). The membranes were soaked in 5% bovine serum albumin (BSA) at 4°C for 8 h and incubated with primary antibody at 4°C overnight. The membranes were washed five times with TBST buffer (50 mM Tris, 150 mM NaCl, 0.05% Tween 20, pH 7.4) for 8 min each time. The membranes were incubated with a secondary antibody at a ratio of 1:10,000 at 4°C for 4 h and then washed five times with TBST buffer for 8 min each time. Signals were detected using the ECL plus kit (GE Healthcare, Piscataway, NJ) following the manufacturer’s specified protocol. All antibodies are listed in Table S3.

### Fluorophore labeling of proteins

Fluorophore labeling of proteins was performed as described with minor modifications (78). Briefly, a cysteine residue was introduced at the C-terminus of the *exsI* sequence, as the ExsI protein lacks cysteine residues. The resulting cysteine-containing ExsI protein was purified and treated with 5 mM dithiothreitol (DTT) for 2 h at room temperature to reduce any potential disulfide bonds. The protein was then dialyzed into the solution containing 20 mM potassium phosphate (pH 7.0) and 500 mM NaCl to remove DTT at 4°C. Next, the dialyzed protein was incubated overnight in the dark at 4°C with 10 mM maleimide fluorophores (Thermo Fisher Scientific, USA, catalog no. A10254) dissolved in dimethyl sulfoxide with reduced protein at a molar ratio of 5:1 (maleimide: protein). A 2 mM DTT was added to stop the reaction. The protein was dialyzed overnight at 4°C into 2 L of 20 mM potassium phosphate (pH 7.0) and 500 mM NaCl.

### Fluorescent labeling of live bacteria

Fluorescent labeling of live bacterial strains was performed as previously described, with minor modifications (78). Briefly, *C. glutamicum* cells in the late exponential phase, grown in LB broth, were collected and washed three times with M9 medium. The cells were resuspended in M9 medium containing 1 µM fluorophore-conjugated protein and incubated at room temperature in the dark for 30 min. The cell suspension was centrifuged, washed at least three times with M9 medium to remove free fluorophore-conjugated protein, and resuspended in M9 medium. The cell suspension was examined using a high-speed rotary disc-type fluorescence confocal microscope (Andor Revolution-XD, UK).

### Bacterial sensitivity assays

Sensitivity assays were performed as previously described, with minor modifications (52). Briefly, *C. glutamicum* strains were cultured in 3 mL LB broth at 30°C overnight to reach the stationary phase. The cultures were then diluted 100-fold into fresh 3 mL LB broth containing 1 mM IPTG and the appropriate antibiotics and grown to the late exponential phase. One milliliter of cells was harvested, washed three times with M9 medium, and then diluted 100-fold into M9 medium containing 15 mM H_2_O_2_ and incubated at 30°C for 40 min with shaking at 100 rpm. Following incubation, the cells were serially diluted and plated onto LB agar plates. The unstressed group served as the control. After 36 h, colonies were counted to calculate the survival percentage. To evaluate the protective effect of exogenous Fe^3+^ against the H_2_O_2_ challenge, 1 mL of *C. glutamicum* cells was harvested, washed, and diluted 100-fold into M9 medium containing 15 mM H_2_O_2_ and 1 µM Fe^3+^. The mixture was incubated at 30°C for 40 min with shaking at 100 rpm. The H_2_O_2_-treated group and unstressed group served as the controls. The remaining steps were performed in the same procedure. To assess the survival of *M. smegmatis* under H_2_O_2_ challenge, 1 mL of *M. smegmatis* cells was harvested, washed, and resuspended in M9 medium containing 5 mM H_2_O_2_. The cells were then incubated at 37°C for 6 h with shaking at 100 rpm. The unstressed group served as the control. After incubation, the cells were serially diluted and plated onto 7H10 agar plates. After 72 h, colonies were counted to calculate the survival percentage. All assays were performed in triplicate and repeated at least three times.

### Intracellular ROS detection

Intracellular ROS detection was carried out as previously described with minor modifications (79). Briefly, 1 mL late-exponential phase *C. glutamicum* cells were collected and washed with M9 medium three times. The cells were resuspended in M9 medium containing 15 mM H_2_O_2_, and then incubated at 30°C for 40 min with shaking at 100 rpm. The unstressed group served as the control. After 40 min, the cells were further washed with M9 medium three times and resuspended in M9 medium containing 10 µM 5- (and-6)-chloromethyl-2′,7′-dichlorodihydrofluorescein diacetate, acetylester (CM-H2DCFDA, Life Technologies, USA) or 3′-(p-hydroxyphenyl) fluorescein (HPF, Invitrogen), and then incubated in the dark for 30 min. After incubation, the cells were collected and washed with M9 medium to remove free fluorescent dye and resuspended in M9 medium. A 200 µL cell suspension was put into a dark 96-well plate. Fluorescence signals were measured using a SpectraMax M2 Plate Reader (Molecular Devices, USA) with excitation/emission wavelengths of 495/520 nm (CM-H2DCFDA) or 490/515 nm (HPF). To test the protective effect of exogenous Fe^3+^ under the H_2_O_2_ challenge on ROS levels, 1 mL late-exponential phase *C. glutamicum* cells were collected and washed with M9 medium three times. The cells were resuspended in M9 medium containing 15 mM H_2_O_2_ and 1 µM Fe^3+^, and incubated at 30°C for 40 min with shaking at 100 rpm. The remaining steps were performed in the same procedure. All these assays were performed in triplicate at least three times.

### Bacterial competition assay

The bacterial competition assay was carried out as previously described with minor modifications (80). Briefly, late-exponential phase *C. glutamicum, B. thailandensis,* and *E. coli* strains were collected and washed with M9 medium three times. *C. glutamicum* (competitor) was mixed with *B. thailandensis* (participant) in M9 medium in a ratio of 10:1. *C. glutamicum* (competitor) was mixed with *E. coli* (participant) in M9 medium in a ratio of 10:1. A total of 100 µL mixtures were then taken out, appropriately diluted, and plated onto LB agar plates. The remaining mixtures were incubated at 30°C (*B. thailandensis*) and 26°C (*E. coli*) with shaking at 130 rpm, respectively. After 12 h of competition, mixtures were appropriately diluted and plated onto LB agar plates. After 24 h, colonies were counted, and the CFU ratio of the competitor to the participant was calculated. 1 µM Fe^3+^ was added to the M9 medium according to the experimental requirement. All these assays were performed in triplicate at least three times.

### Determination of intracellular iron content

Bacterial intracellular iron content was determined as described previously with minor modifications (81, 82). Briefly, 10 mL late-exponential phase *C. glutamicum* cells were collected and washed with M9 medium three times. The cells were resuspended with 10 mL M9 medium containing 15 mM H_2_O_2_ and 1 µM Fe^3+^, and incubated at 30°C for 20 min with shaking at 100 rpm. After incubation, the cells were further washed with M9 medium and resuspended in Bugbuster solution (Novagen, Madison, WI) on a rotator at 4°C for 30 h. The cell suspension was centrifuged at 13,300 rpm for 40 min at 4°C. Protein concentrations were determined by the Bradford assay with BSA as a standard. Each supernatant was diluted fivefold with 2% molecular-grade nitric acid to a total volume of 4 mL. The supernatant was then incubated on a rotator at room temperature for 24 h, followed by centrifugation at 10,000 rpm for 1 h. The samples were measured using atomic absorption spectrometry analysis (PinAAcle 900F, USA). Triplicate cultures of each strain were analyzed during a single experiment, and the experiment was repeated at least three times.

### Electrophoretic mobility shift assay

EMSAs were performed as described previously with minor modifications (83, 84). Briefly, the genomic DNA of *C. glutamicum* was used as a template to amplify the T7SS promoter fragment by PCR. Then the T7SS promoter fragment was purified and quantified. For the DNA-binding assay, the T7SS promoter fragment was incubated with Fur protein in a total volume of 20 µL EMSA buffer at room temperature for 30 min. The binding reaction mixtures were separated by 6% native PAGE gels containing 5% glycerol, and electrophoresis was performed at 100 V and 4°C with 0.5 Tris-borate-EDTA buffer. Gels were stained with SYBR Safe DNA Gel Stain (Invitrogen, USA), and images were acquired using the Universal Hood II system (Bio-Rad Laboratories, Inc.). DNA-binding assay reaction system: 2 µL 10× binding buffer, 1 µL 1 mM KCl, 1 µL 20 mM EDTA, 1 µL 50% glycerol, 90 ng T7SS promoter fragments, Fur proteins (0, 0.5, 1, 2, 4, 8, 14 µg), and the remaining volume is filled up to 20 µL with ultra-pure water. 10× binding buffer: 100 mM Tris, 500 mM KCl, 10 mM DTT; pH 7.5, stored at –20°C. The DNA fragment sequence with a scrambled is “AGTCGGGCGTTGCTCCTCTATATCGACCTAGAGTCGCCTAACCTGCTTCTCTCAAAATAGATAAGGGGAGCGCAAGAGGTATAATTCTCCGCCTTATAAGGCATAACTGCGTCTAAACGGGGTGTTAACTATAAAAAGCATTTTCGATCGTCAAAGTGAAGTCCGAAGAGGGAACGGGCTACACAACTTTTGGATTTTCGACTGCGAGTTGCGTTGGGAGAGGGCTTTTACGTCTCGCAATACAACGTCGCGTTCGAGTTGGCCTCAATCGTCGACAGATAGTCTTCGACCGCTACGTCTATCTAACCCTGCTTATTTGGTGCTTGCAAGGCGATTCATTATGCGTCCAG.”

### Ferene S staining assay

The Ferene S staining assay was carried out as previously described with minor modifications (85, 86). Briefly, the purified protein was resuspended in a pH 8.6 buffer containing 0.1 M sodium citrate and 0.1 M NaHCO_3_. Different concentrations of Fe^3+^ were added to the suspension and incubated at room temperature for 10 min. The proteins were then dialyzed with a pH 8.0 buffer containing 10 mM Tris and 200 mM NaCl to remove free Fe^3+^. The resulting protein was mixed with the stain at a ratio of 1:1 in a volume, and the reaction was carried out at room temperature for 5 min. The protein was combined with the stain, dot blotted onto a nitrocellulose membrane, and observed for a blue color. The stain consisted of 0.75 mM Ferene S, 2% glacial acetic acid, and 15 mM thioglycolic acid.

### Intracellular survival analysis

Intracellular survival analysis was performed as described previously with minor modifications (87). Briefly, RAW264.7 cells were seeded into 24-well plates at a density of 3 × 10^5^ cells/well and incubated overnight for adhesion. *M. smegmatis* wild-type and Δ*exsI^ms^* strains were then used to infect the RAW264.7 cells at a multiplicity of infection of 10:1 for 4 h. Extracellular bacteria were eliminated through the addition of fresh medium containing 100 µg mL^–1^ gentamicin. At 6 and 24 h post-infection, 0.1% Triton X-100 was used to lyse the infected cells. The lysates were diluted with PBS buffer and then plated onto 7H10 agar plates, and colonies were counted after 3 days. All samples were analyzed in triplicate.

### The growth curve

*C. glutamicum* strains were cultured in LB broth at 30°C overnight to reach the stationary phase and then diluted 100-fold into fresh LB broth containing 1 mM IPTG and the appropriate antibiotics. The strains were cultured at 30°C with shaking at 220 rpm. The growth of strains was monitored by determining OD_600_ at the specified time points. The different culture conditions are outlined as follows: LB medium; LB medium containing 70 µM EDDHA; LB medium containing 70 µM EDDHA and 15 mM H_2_O_2_.

### Analysis of the subcellular location of ExiR in *C. glutamicum*

Briefly, a fluorescence-based assay using GFP-conjugated ExiR was performed to identify its location *in vivo*. Plasmid pXMJ19-*gfp-exiR* was transformed into *C. glutamicum* wild type by electroporation. The resulting strain was grown in LB broth until the late-exponential phase. One milliliter of cells was harvested, washed with M9 medium three times, and resuspended in M9 medium. The cell suspension was observed by a high-speed rotary disc-type fluorescence confocal microscope (Andor Revolution-XD, UK).

Protein extraction was performed as described previously with minor modifications (88). Briefly, the plasmids pXMJ19-*exiR-vsvg* and pXMJ19-*ftsH-vsvg* were transformed into *C. glutamicum* Δ*exiR* mutant via electroporation, respectively. A total of 50 mL of the resulting strains wase harvested, washed three times with PBS buffer, and then resuspended in PBS buffer containing 0.2 mM phenylmethanesulfonyl fluoride. The cells were lysed, and the supernatant was centrifuged at 8,000 rpm for 10 min at 4°C to remove unlysed cells. The supernatant was then collected and centrifuged at 105,000 × *g* for 1.5 h at 4°C. Following this centrifugation step, the supernatants were carefully collected and served as the cytoplasmic fractions. The remaining protein pellets were resuspended in PBS buffer containing 1% Triton X-100 and then solubilized overnight at 4°C with gentle agitation on a rotator. The solutions were centrifuged at 16,000 × *g* for 15 min at 4°C, and the supernatants were collected to serve as the membrane fractions. All cell fraction samples were separated by SDS-PAGE and detected using western blot analysis.

### Iron binding prediction

The tertiary structures of ExsI (Cgl0579) and Cgl0580 were calculated via the AlphaFold3 (https://alphafoldserver.com/) based on the amino acid sequence of the protein (48). The structure of Fe^3+^ was obtained from PubChem (https://pubchem.ncbi.nlm.nih.gov/). Docking simulations were performed with AutoDock Vina 1.2.5, and the best binding mode was selected based on the lowest docking energy (49, 50). The three-dimensional figures were displayed using PyMOL (https://pymol.org/).

### Amino acid sequence alignment and structure prediction of ExsI

Amino acid sequence search was carried out using BLAST programs at the BLAST server of the National Center for Biotechnology Information (NCBI) website (https://www.ncbi.nlm.nih.gov/), and multiple sequence alignment of ExsI and Fur homologs was performed using DNAMAN 8.0 software. The structural model of ExsI was generated using I-TASSER (https://zhanggroup.org//I-TASSER/).

### Bioinformatic surveys and phylogenetic analysis

Search for ExsI homologs was performed using the BLASTP program against the NCBI non-redundant protein database with a cutoff E-value of 1E-06 and a coverage threshold of 60%. The resulting ExsI homologs are listed in Supplementary Data 2. For high-confidence ExsI homologs that could be unambiguously assigned to order level, one representative sequence for each order was randomly selected and subjected to phylogenetic analysis. Multiple protein sequence alignments were performed with MEGA 11.0 software, and phylogenetic trees were constructed with MEGA 11.0 software using the maximum-likelihood method based on the Jones-Taylor-Thornton model. Bioinformation surveys for ExiR homologs and phylogenetic analysis were performed using the same method, and the resulting ExiR homologs are listed in Supplementary Data 3.

### Enzyme-linked immunosorbent assay

Calprotectin concentrations in serum were assessed using a CALP ELISA kit (SHANGHAI HUDING BIOTECHNOLOGY), following the manufacturer’s instructions. Whole blood was stored in plastic serum separation tubes and incubated for 1 h at 37°C, followed by centrifugation at 2,300 × *g* for 5 min to obtain clear serum. Clear serum was transferred to new tubes and stored at –80°C for further detection assays.

### Statistical analysis

Statistical analyses of bacterial sensitivity assays, intracellular ROS detection, determination of intracellular iron content, qRT-PCR data, bacterial competition assay, and intracellular survival analysis were performed using a two-tailed Student’s *t*-test for paired comparisons or one-way analysis of variance (ANOVA) with Tukey’s multiple comparisons test. *P* values in mice survival were analyzed using the log-rank (Mantel-Cox) test. *P* values in bacterial CFU were analyzed using the Mann-Whitney test. Statistical analyses were performed using GraphPad Prism Software (GraphPad Software 8.0) with adjusted *P* < 0.05 considered statistically significant.

The rest of the Materials and Methods are described in detail in Text S1 Supplementary Materials and Methods file.

## Data Availability

All data needed to evaluate the conclusions in the paper are present in the paper and/or the Supplementary Materials.

## SUPPLEMENTAL MATERIAL

Supplemental figures mbio.02419-25-s0001.docx Fig. S1 to S9.

Data S1 mbio.02419-25-s0002.xlsx Mass spectrometry peptide identification results.

Data S2 mbio.02419-25-s0003.xlsx ExsI homologs with a cutoff *E*-value of 1E−06 and a coverage threshold of 60%.

Data S3 mbio.02419-25-s0004.xlsx ExiR homologs with a cutoff *E*-value of 1E−06 and a coverage threshold of 60%.

Text S1 mbio.02419-25-s0005.docx Supplemental materials and methods.

Supplemental tables mbio.02419-25-s0006.docx Tables S1 to S3.

ASM does not own the copyrights to Supplemental Material that may be linked to, or accessed through, an article. The authors have granted ASM a non-exclusive, world-wide license to publish the Supplemental Material files. Please contact the corresponding author directly for reuse.
