# Supplementary material for: Type VII secretion system deploys an active iron uptake pathway to enhance bacterial fitness and counteract host nutritional immunity
Source: mBio. 2025 Sep 25;16(11):e02419-25. doi: 10.1128/mbio.02419-25 (PMC12607788; doi:10.1128/mbio.02419-25)
Supplement: Supplemental figures — Fig. S1 to S9. [file mbio.02419-25-s0001.docx]

**Supplementary Figures**


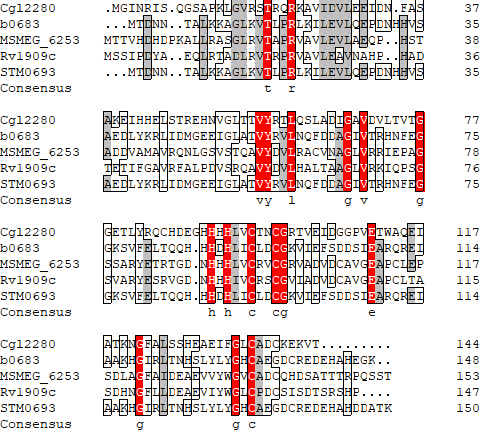


**Fig S1** Fur in different bacterial species. Sequence alignment of *C.glutamicum* Fur protein with Fur protein from other species. The red background showed the strongly conserved residues. The NCBI-ProteinID of these Fur proteins in *C. glutamicum* (BAB99673), *E. coli* (NP_415209), *M. smegmatis* (ABK74212), *M. tuberculosis* (NP_216425) and *S.* Typhimurium (NP_459678).


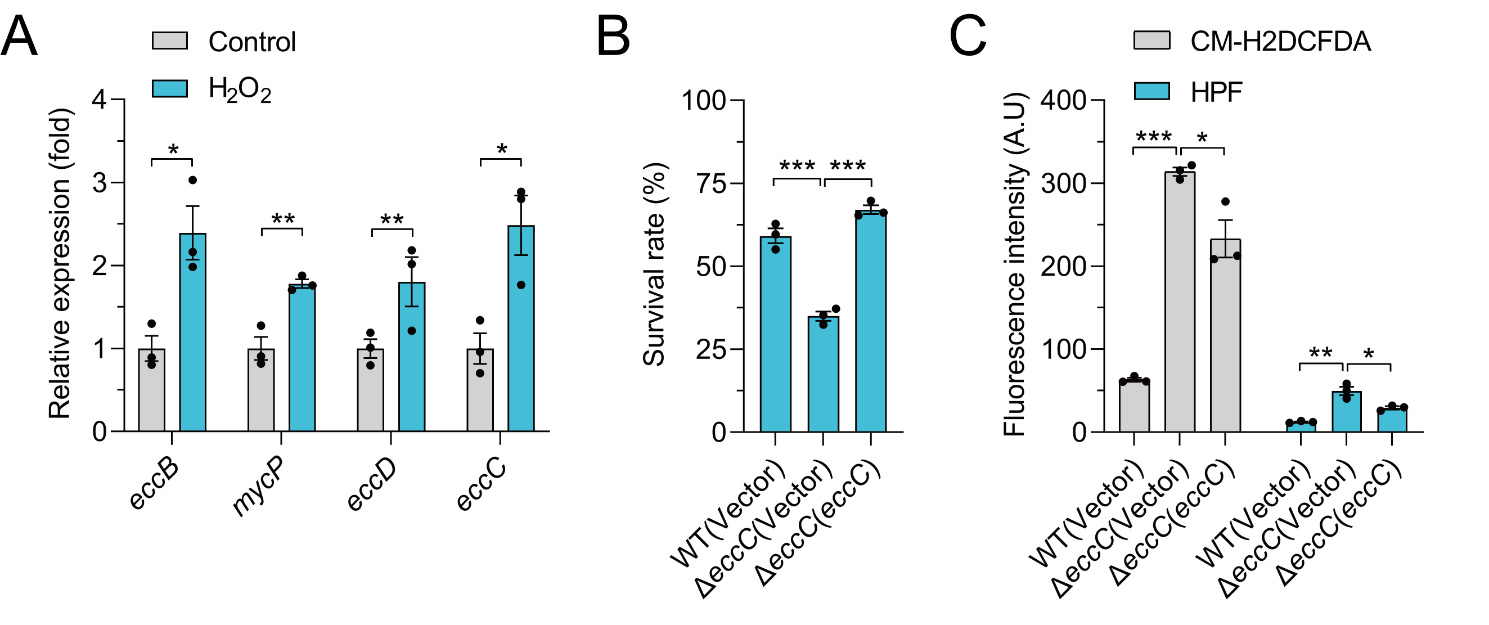


**Fig S2** *C. glutamicum* T7SS contributes to resistance to oxidative stress. (A) Oxidative stress induced the expression of T7SS. *C. glutamicum* WT strains were exposed to 15 mM H_2_O_2_ for 5 min. (B) Relevant late-exponential phase bacterial strains were cultured in M9 medium containing 15 mM H_2_O_2_ for 40 min and the viability of the cells was determined. (C) Intracellular ROS levels in the late-exponential phase *C. glutamicum*, following exposure to H_2_O_2_, were assessed using CM-H2DCFDA and HPF dyes. The fluorescence signals were quantified with a SpectraMax M2 Plate Reader. Data in all panels are mean ± SEM of three independent experiments. **P* < 0.05; ***P* < 0.01; ****P* < 0.001.


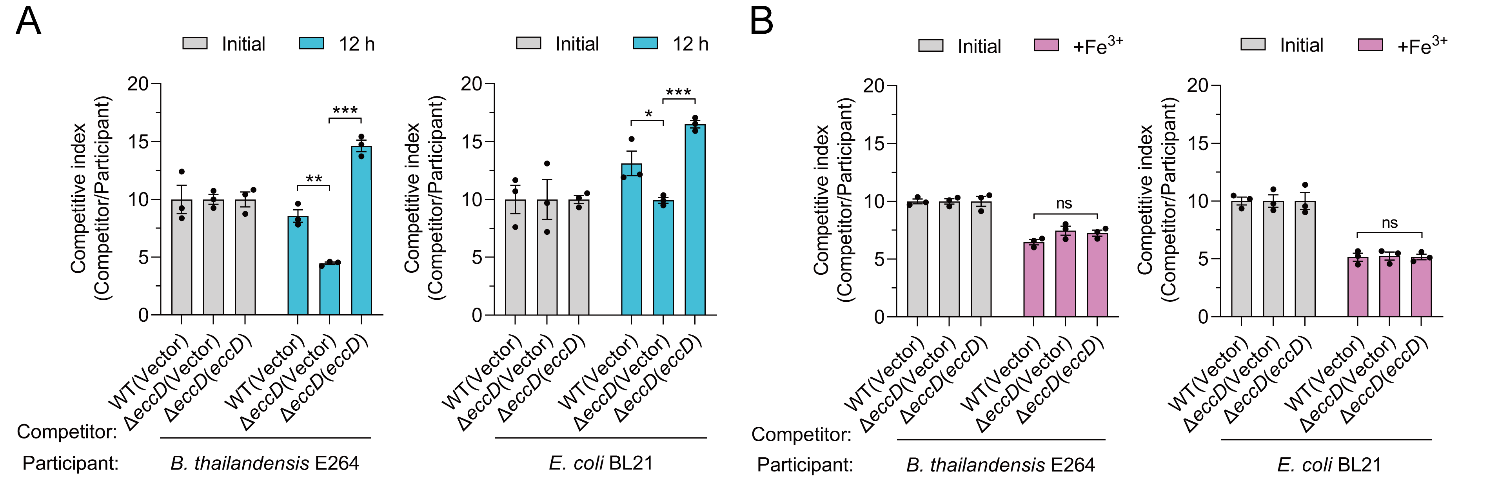


**Fig S3** T7SS plays a significant role in *C. glutamicum*’s competitive advantage over other bacteria. (A) Bacterial competition between *C. glutamicum* and *B. thailandensis* or E*. coli*. *C. glutamicum* (competitor) was mixed with *B. thailandensis* (participant) in M9 medium in a ratio of 10:1 and grown at 30 °C for 12 h (left). *C. glutamicum* (competitor) was mixed with *E. coli* (participant) in M9 medium in a ratio of 10:1 and grown at 26 °C for 12 h (right). Bars represent the mean competitor: participant CFU ratios of three independent experiments (± SEM). (B) Bacterial competition between *C. glutamicum* and *B. thailandensis* or *E. coli*. *C. glutamicum* (competitor) was mixed with *B. thailandensis* (participant) in M9 medium containing 1 μM Fe^3+^ in a ratio of 10:1 and grown at 30 °C for 12 h (left). *C. glutamicum* (competitor) was mixed with E*. coli* (participant) in M9 medium containing 1 μM Fe^3+^ in a ratio of 10:1 and grown at 26 °C for 12 h (right). Bars represent the mean competitor: participant CFU ratios of three independent experiments (± SEM). **P* < 0.05; ***P* < 0.01; ****P* < 0.001; ns, not significant.


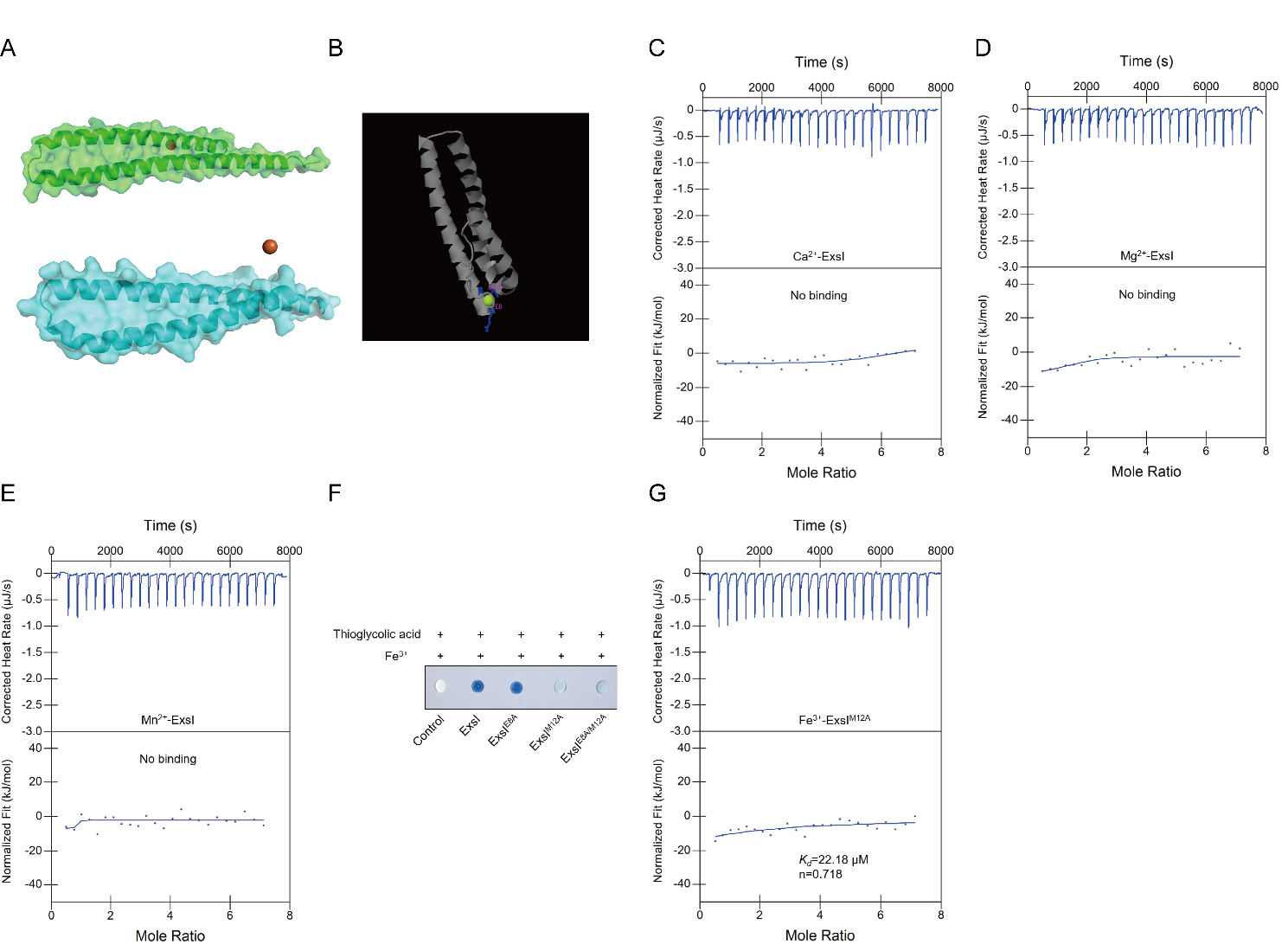


**Fig S4** ExsI is an iron-binding protein. (A) Surface and cartoon representations of the structural models of Cgl0579 (Exsl) (top) and Cgl0580 (bottom), each docked with Fe^3+^, were generated using PyMOL. Fe^3+^ is shown as a brown sphere. The 3D structures of Cgl0579 and Cgl0580 were predicted using Alphafold3. (B) The structure model of ExsI binding to ligand predicted by I-TASSER. The green ligand represents Fe^3+^, and the ligand binding site residues are Glu8 and Met12. (C-E) The ability of binding Ca^2+^ (C), Mg^2+^ (D), and Mn^2+^ (E) by ExsI was evaluated using isothermal titration calorimetry (ITC). Data were analyzed using the Nano Analyze software (TA Instruments). (F) The ability to bind Fe^3+^ by ExsI mutant proteins was determined by the Ferene S staining assay. The proteins were mixed with the stain, dot-blotted onto a nitrocellulose membrane, and then observed for the development of a blue color. (G) The ability of binding Fe^3+^ by ExsI^M12A^ was evaluated using isothermal titration calorimetry (ITC). Data were analyzed using the Nano Analyze software (TA Instruments). (C-G) Representative images from three independent experiments.


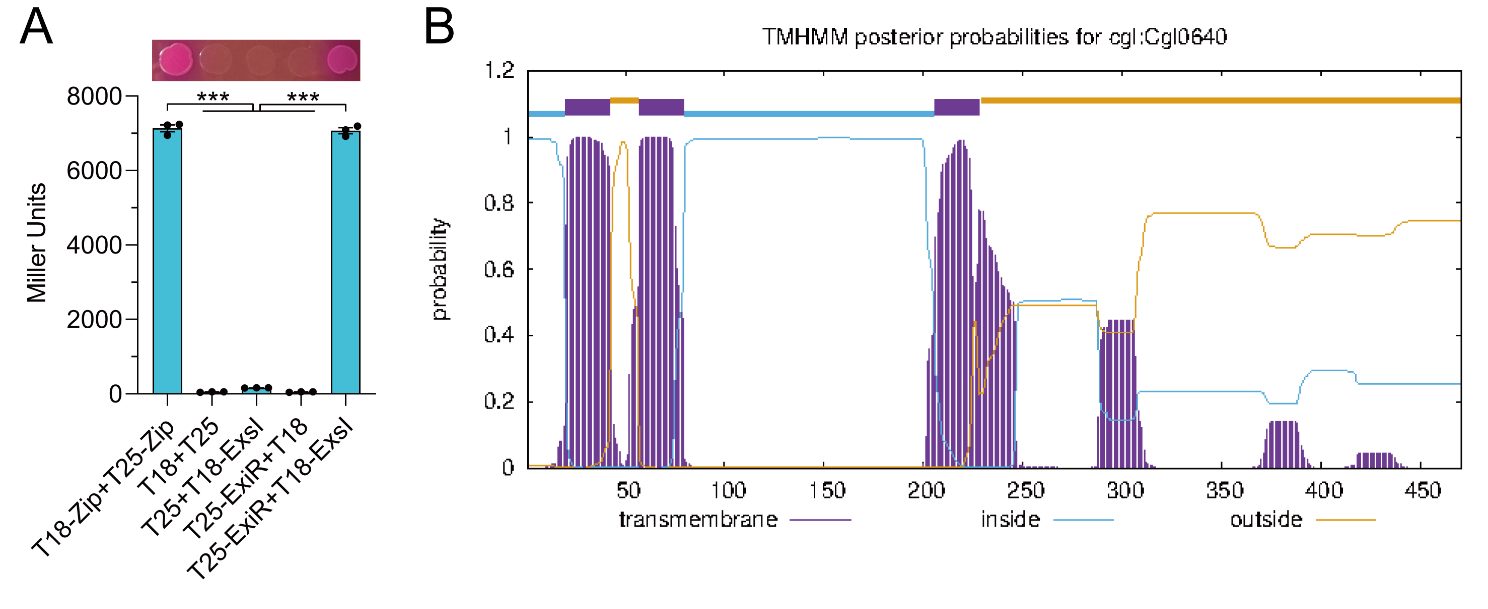


**Fig S5** ExsI and ExiR exhibit direct interactions. (A) Interactions between ExsI and ExiR were assessed using MacConkey maltose plates (upper) and the β-galactosidase assay (lower). Data are mean ± SEM of three independent experiments. ****P* < 0.001. (B) The prediction of transmembrane helices in ExiR using the online website TMHMM2.0.


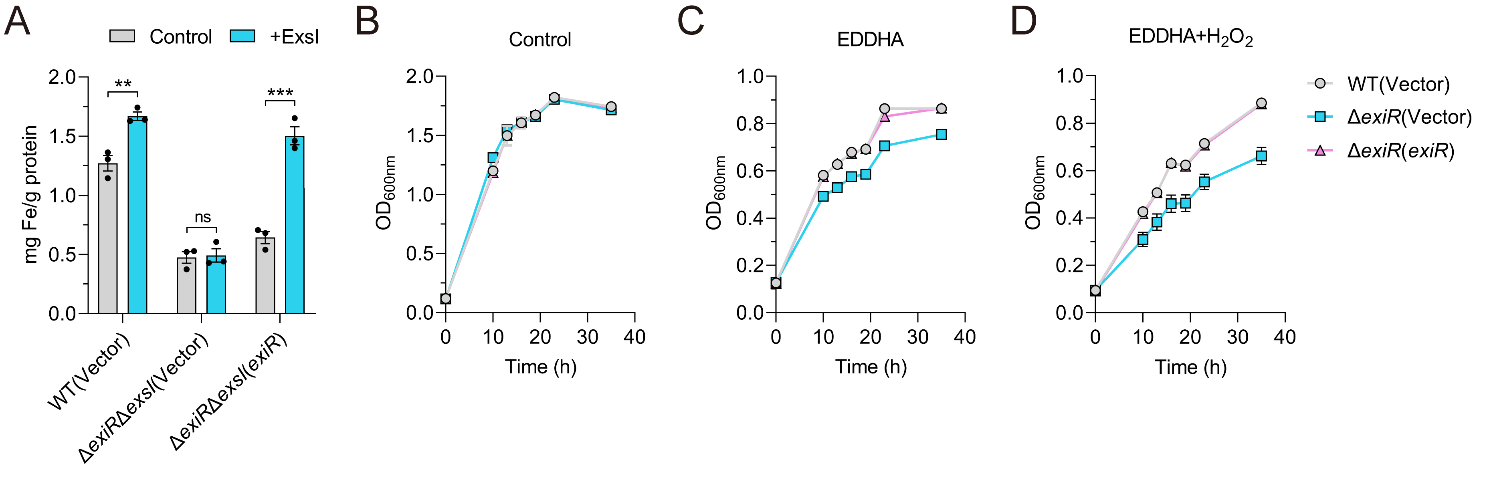


**Fig S6** ExiR is required for the growth and Fe^3+^ import of *C.glutamicum* strains under Fe^3+^-limited and oxidative stress conditions. (A) Relevant late-exponential phase bacterial strains were exposed to 15 mM H_2_O_2_ with the addition of 1 μM Fe^3+^ for 20 min, supplemented with or without 1 μM ExsI protein. Iron associated with bacterial cells was measured by atomic absorption spectrometry analysis. (B-D) The relevant *C. glutamicum* strains grown overnight were harvested and diluted 100-fold into different mediums: LB medium (B), LB medium supplemented with 70 μM EDDHA (C), LB medium containing 70 μM EDDHA and 15 mM H_2_O_2_ (D). The cultures were incubated at 30 °C, and their growth was monitored by measuring the OD_600_ at specified time points. Data in all panels are mean ± SEM of three independent experiments.


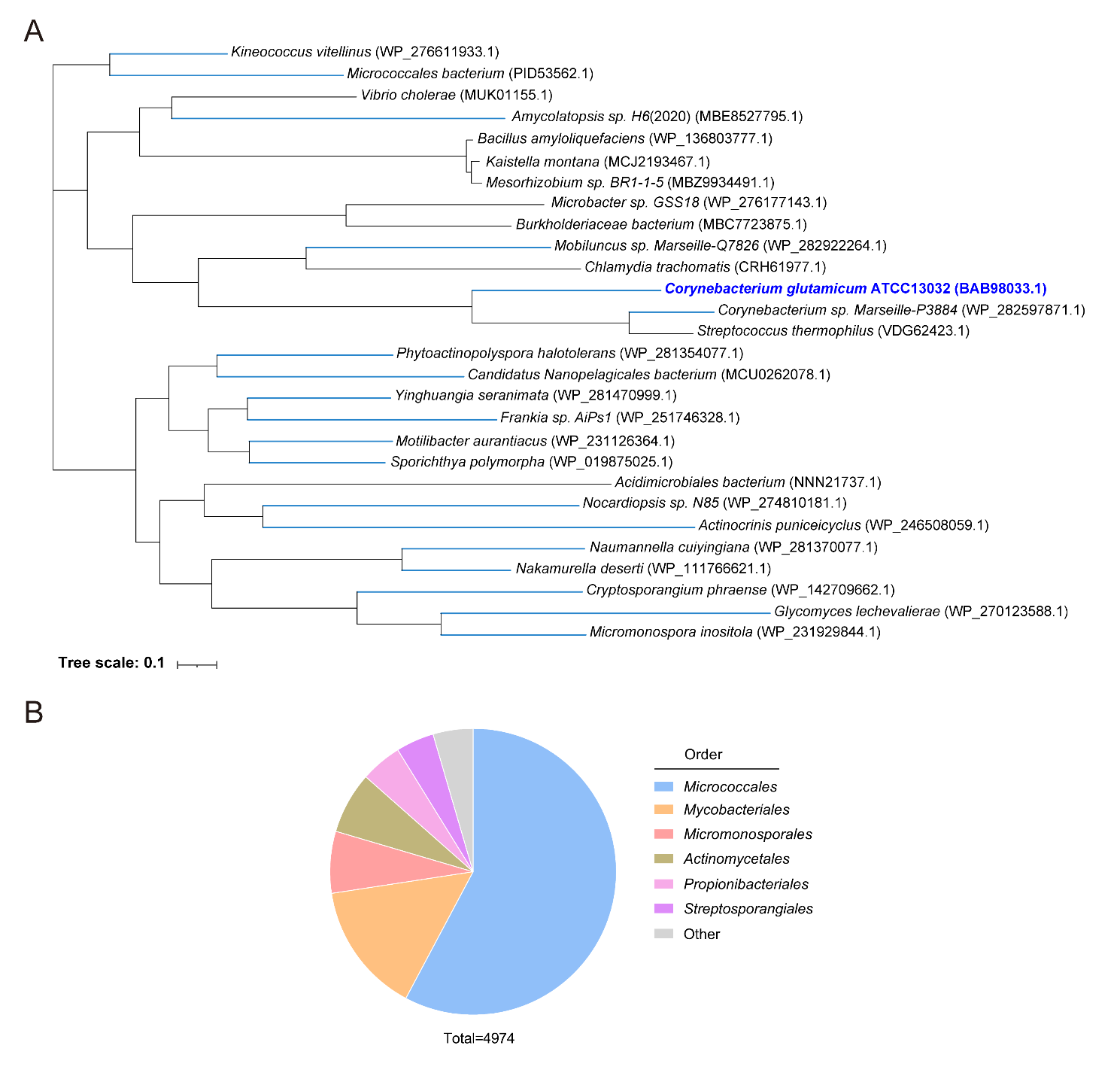


**Fig S7** ExiR homologs are widespread and conserved in different bacterial species. (A) Maximum-likelihood phylogenetic tree showing the relationship of the ExiR from *C.glutamicum* and 27 high-confidence ExiR orthologs from other genes. High-confidence ExiR homologs were searched by BLASTP. One representative sequence for each order was randomly selected and multiple sequence alignments were performed with MEGA 11.0 software. The phylogenetic tree was reconstructed by using the maximum-likelihood method in the MEGA 11.0 software. The bacterial species to which ExiR homologs belong are provided and the NCBI accession number for each ExiR homolog is given in parentheses. Bootstrap values (expressed as percentages of 1000 replications) are shown on the branch. The scale bar indicates an evolutionary distance of 0.1 amino acid substitutions per position. (B) Percentage distribution of 4974 high-confidence ExiR homologs at the order level in prokaryotes.


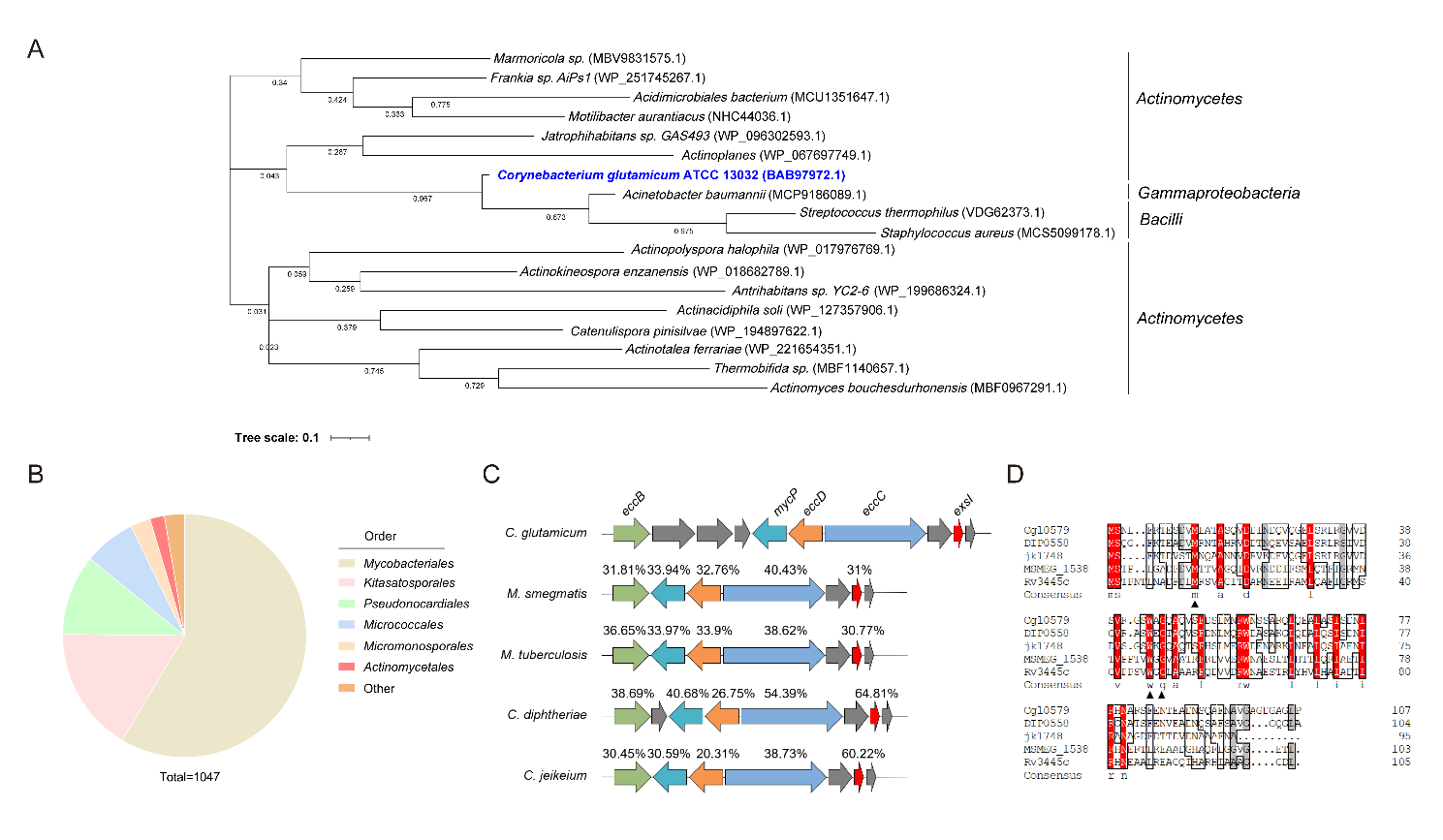


**Fig S8** ExsI homologs are widespread and conserved in different bacterial species. (A) Maximum-likelihood phylogenetic tree showing the relationship of the ExsI from *C.glutamicum* and 17 high-confidence ExsI orthologs from other genes. High-confidence ExsI homologs were searched by BLASTP. One representative sequence for each order was randomly selected and multiple sequence alignments were performed with MEGA 11.0 software. The phylogenetic tree was reconstructed by using the maximum-likelihood method in the MEGA 11.0 software. The bacterial species to which ExsI homologs belong are provided and the NCBI accession number for each ExsI homolog is given in parentheses. Bootstrap values (expressed as percentages of 1000 replications) are shown on the branch. The scale bar indicates an evolutionary distance of 0.1 amino acid substitutions per position. (B) Percentage distribution of 1047 high-confidence ExsI homologs at the order level in prokaryotes. (C) Comparison of T7SS cluster amino acid sequences from different species. The similarity is shown. NCBI-ProteinID: *C. glutamicum* (BAB97964; BAB97968; BAB97969; BAB97970; BAB97972); *M. smegmatis* (ABK73967; ABK69620; ABK71110; ABK72753; ABK74158); *M. tuberculosis* (NP_217967; NP_217966; NP_217965; NP_217964; NP_217962); *C. diphtheriae* (CAE49064; CAE49066; CAE49067; CAE49068; CAE49070); *C. jeikeium* (CAI37930; CAI37929; CAI37928; CAI37927; CAI37925). (D) Sequence alignment of ExsI with homologs from other species using DNAMAN 8.0 software. The red background showed the strongly conserved residues. The black triangle represents the conserved Met12 amino acid and the WXG motif. NCBI-ProteinID: *Cgl0579* (BAB97972); *MSMEG_1538* (ABK74158); *Rv3445c* (NP_217962); *DIP0558* (CAE49070); *jk1748* (CAI37925).


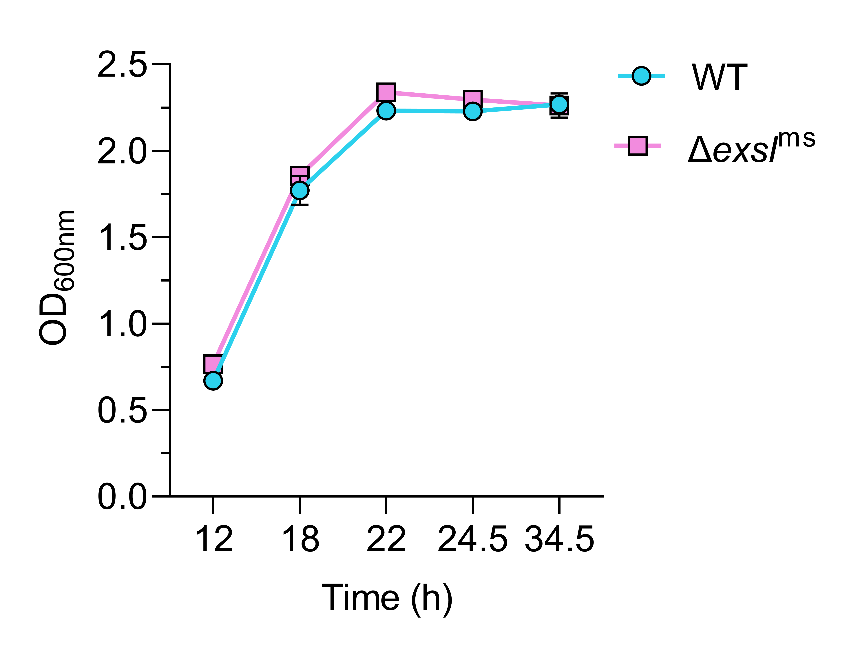


**Fig S9** Deletion of the *exsI^ms^* gene does not affect the growth of the *M. smegmatis* strain under normal conditions. The growth of the relevant strains was detected by measuring OD_600_ at indicated time points.
