# Supplementary material for: Type VII secretion system deploys an active iron uptake pathway to enhance bacterial fitness and counteract host nutritional immunity
Source: mBio. 2025 Sep 25;16(11):e02419-25. doi: 10.1128/mbio.02419-25 (PMC12607788; doi:10.1128/mbio.02419-25)
Supplement: Text S1 — Supplemental materials and methods. [file mbio.02419-25-s0005.docx]

# Supplementary Materials and Methods

## Plasmid construction

Primers used in this study are listed in Supplementary Table 2. To construct the knock-out plasmid for deletion of *exsI* in *C. glutamicum*, the 982 bp upstream fragment and the 746 bp downstream were amplified using the primer pairs D*exsI*-F1/D*exsI*-R1 and D*exsI*-F2/D*exsI*-R2, respectively. The upstream and downstream PCR fragments were ligated by overlap PCR, and the resulting PCR product was inserted into the *Eco*RI/*Hin*dIII sites of the suicide vector pK18*mobsacB* to produce pK18*mobsacB*-Δ*exsI*. The knock-out plasmids pK18*mobsacB*-Δ*eccD* (*Cgl0576*), pK18*mobsacB*-Δ*exiR* (*Cgl0640*) and pK18*mobsacB*-Δ*fur* (*Cgl2280*) were obtained with the same method. For complementation to Δ*exsI* mutant, the primer pairs *exsI*-F1/*exsI*-R1 were used to amplify the *exsI* gene from the genomic DNA of *C. glutamicum*. The resulting *exsI* fragment was inserted into the *Hin*dIII/*Bam*HI sites of pXMJ19 to produce the complementation plasmid pXMJ19-*exsI*-*vsvg*. The complementation plasmids pXMJ19-*eccD*-*vsvg*, pXMJ19-*eccC*-*vsvg*, pXMJ19-*fur*, pXMJ19-*exiR*-*vsvg*, pXMJ19-*MSMEG_1538*-*vsvg*, pXMJ19-*Rv3445c*-*vsvg*, pXMJ19-*jk1748*-*vsvg*, pXMJ19-*DIP0558*-*vsvg*, and pXMJ19-*ftsH*-*vsvg* (*Cgl2696*) were obtained with the same method. Plasmid pXMJ19-*exsI*-*vsvg* was also constructed for protein secretion assay. To construct the expressing plasmid pET28a-*exsI*, the primer pairs *exsI*-F2/*exsI*-R2 were used to amplify the *exsI* gene from the genomic DNA of *C. glutamicum*. The resulting *exsI* fragment was inserted into the *Bam*HI/*Hin*dIII sites of pET28a to produce the expressing plasmid pET28a-*exsI*. The expressing plasmids pET28a-*exsI^M12A^*, pET28a-*exsI*-*Cys*, pET28a-*exsI^E8A/M12A^*, pET28a-*exsI^E8A^* and pET28a-*fur* were obtained with the same method. To construct the expressing plasmid pGEX-6p-1-*exsI*, the primer pairs *exsI*-F3/*exsI*-R3 were used to amplify the *exsI* gene from the genomic DNA of *C. glutamicum*. The resulting *exsI* fragment was inserted into the *Sal*I/*Bam*HI sites of pGEX-6p-1 to produce the expressing plasmid pGEX-6p-1-*exsI*. To construct the expressing plasmid pET21aHMT-*Rv3445c*, the primer pairs *Rv3445c*-F2/*Rv3445c*-R2 were used to amplify the *Rv3445c* gene from the genomic DNA of *M. tuberculosis*. The resulting *Rv3445c* fragment was digested and inserted into the *Eco*RI/*Xho*I sites of pET21aHMT to produce the expressing plasmid pET21aHMT-*Rv3445c*. The expressing plasmids pET21aHMT-*MSMEG_1538*, pET21aHMT-*jk1748* and pET21aHMT-*DIP0558* were obtained with the same method. For constructing plasmids used in bacterial two-hybrid assays, the *exsI* and *exiR* genes for testing were amplified by PCR from the genomic DNA of *C. glutamicum* using *exsI*-F6/*exsI*-R6 and *exiR*-F2/*exiR*-R2. Amplified DNA fragments were cloned into the corresponding sites of pKT25 and pUT18C vectors, respectively. For constructing the pXMJ19-*gfp*-*exiR*, the 720 bp *gfp* fragment and the 1416 bp *exiR* fragment were amplified using the primer pairs *gfp*-*exiR*-UP-F/*gfp*-*exiR*-UP-R and *gfp*-*exiR*-DOWN-F/*gfp*-*exiR*-DOWN-R, respectively. The upstream and downstream PCR fragments were ligated by overlap PCR, and the resulting PCR product was inserted into the *Hin*dIII/*Bam*HI sites of pXMJ19 to produce pXMJ19-*gfp*-*exiR*. For constructing the pMV261-*exsI^ms^* (*MSMEG_1538*), the primer pairs *exsI^ms^*-F1/*exsI^ms^*-R1 were used to amplify the *exsI^ms^* gene from the genomic DNA of *M. smegmatis*. The resulting *exsI^ms^* fragment was digested and inserted into the *Eco*RI/*Hin*dIII sites of pMV261 to produce the plasmid pMV261-*exsI^ms^*. For constructing the pYC1240-*sgrna*, the primer pairs *sgrna*-F/*sgrna*-R were used to amplify the 25 bp fragment. The resulting fragment was inserted into the *Bpm*I/*Hin*dIII sites of pYC1240 to produce the plasmid pYC1240-*sgrna*. For constructing the pEC-XK99E-*eccD*, the primer pairs *eccD*-F2/*eccD*-R2 were used to amplify the *eccD* gene from the genomic DNA of *C. glutamicum*. The resulting *eccD* fragment was digested and inserted into the *Kpn*I/*Sal*I sites of pEC-XK99E to produce the plasmid pEC-XK99E-*eccD*. All the plasmid derivatives were confirmed by PCR and DNA sequencing.

## In-frame deletion and complementation in *C. glutamicum*

The in-frame deletion was performed as previously described with minor modifications(1-3). Briefly, for constructing in-frame deletion mutants, the suicide plasmid pk18*mobsacB* derivatives were transformed into *C. glutamicum* competent cells through electroporation, followed by incubating at 46 °C immediately for 6 min without shaking. The transformants were selected by plating on LB agar plates supplemented with nalidixic acid (20 μg mL^–1^) and kanamycin (50 μg mL^–1^). Then the successful transformant was cultured in LB broth containing nalidixic acid (20 μg mL^–1^) at 30 °C. After 16 h, 5 μL cultures were plated on LB agar plates supplemented with nalidixic acid (20 μg mL^–1^) and 20 % sucrose. All successful mutants were verified correctly by PCR and sequencing. For complementation, the pXMJ19 derivatives were transformed into relevant mutant strains by electroporation.

## In-frame deletion and complementation in *M. smegmatis*

To delete the *exsI^ms^* (*MSMEG_1538*) gene in *M. smegmatis*, we used the CRISPR Cas12a-assisted recombineering method(4). Briefly, the genomic DNA of *M. smegmatis* was used as the template to amplify the upstream and downstream of the *exsI^ms^* gene by PCR, then the upstream and downstream PCR fragments were ligated by overlap PCR. pYC1240 plasmid carrying the sequence adjacent to 5’-TTN-3’ was constructed. Both the pYC1240 derivative and the recombined fragments were co-transformed into *M. smegmatis* containing the pYC917 plasmid, which expresses the Cas12a protein, via electroporation at 2.5 kV. After electroporation, 1 mL 7H9 broth was added and cultured at 30 °C for 2 h. The mutant colonies were selected by plating on 7H10 agar plates supplemented with hygromycin (50 μg mL^–1^), kanamycin (50 μg mL^–1^) and anhydrotetracycline (100 ng mL^–1^). The Δ*exsI^ms^* mutant was verified correctly by PCR and sequencing. For complementation, the pMV261-*exsI^ms^* was transformed into Δ*exsI^ms^* mutant by electroporation.

## Overexpression and purification of recombinant proteins

To express His_6_-, GST-, or MBP-tagged recombinant proteins, corresponding derivatives of pET28a, pGEX-6p-1, or pET21aHMT were transformed into *E. coli* BL21(DE3) cells, with heat treatment at 42 °C for 90 s, respectively. The resulting strains were cultured in 3 mL LB broth at 37 °C overnight and diluted 100-fold into fresh LB broth. The strains were cultured at 37 °C until an OD_600_ of 0.4, and induced with 0.3 mM isopropylb-D-1-thiogalactopyranoside (IPTG) at 22 °C overnight. The cells were harvested and resuspended with the corresponding buffer (20 mM Tris-HCl, 0.5 M NaCl, pH 7.4) at 4 °C, and broken by sonification. The proteins were purified using GST•Bind resin, His•Bind nickel-nitrilotriacetic acid resin, and MBP•Bind resin (Novagen, Madison, WI), according to the manufacturer’s instructions. Purified recombinant proteins were dialyzed against the appropriate buffer overnight at 4 °C, and were analyzed by SDS–PAGE. The recombinant proteins were stored at –80 °C until use. For the Ferene S staining assay, the MBP tag was removed by incubation with protease at 4 °C overnight. Separate proteins through MBP•Bind resin and analysis by SDS–PAGE.

# References

1. Schäfer A, Tauch A, Jäger W, Kalinowski J, Thierbach G, Pühler A. 1994. Small mobilizable multi-purpose cloning vectors derived from the *Escherichia coli* plasmids pK18 and pK19: selection of defined deletions in the chromosome of *Corynebacterium glutamicum.* Gene 145:69-73. <http://doi.org/10.1016/0378-1119(94)90324-7>

2. van der Rest M E, Lange C, Molenaar D. 1999. A heat shock following electroporation induces highly efficient transformation of *Corynebacterium glutamicum* with xenogeneic plasmid DNA*.* Appl Microbiol Biotechnol 52:541-5. <http://doi.org/10.1007/s002530051557>

3. Tauch A, Kirchner O, Loffler B, Gotker S, Puhler A, Kalinowski J. 2002. Efficient electrotransformation of *corynebacterium diphtheriae* with a mini-replicon derived from the *Corynebacterium glutamicum* plasmid pGA1*.* Curr Microbiol 45:362-7. <http://doi.org/10.1007/s00284-002-3728-3>

4. Yan M Y, Yan H Q, Ren G X, Zhao J P, Guo X P, Sun Y C. 2017. CRISPR-Cas12a-assisted recombineering in bacteria*.* Appl Environ Microbiol 83. <http://doi.org/10.1128/AEM.00947-17>
