# Supplementary material for: Type VII secretion system deploys an active iron uptake pathway to enhance bacterial fitness and counteract host nutritional immunity
Source: mBio. 2025 Sep 25;16(11):e02419-25. doi: 10.1128/mbio.02419-25 (PMC12607788; doi:10.1128/mbio.02419-25)
Supplement: Supplemental tables — Tables S1 to S3. [file mbio.02419-25-s0006.docx]

**Supplementary Tables**

**Table S1. Bacterial strains, cells, mice, and plasmids used in this study.**

| **Strain or plasmid** | **Relevant characteristics** | **Reference** |
| --- | --- | --- |
| **Bacterial strains** | | |
| ***E. coli*** |  |  |
| BL21(DE3) | Host for expression vector pET21aHMT, pET28a, pGEX-6p-1 and used it for bacterial competition assay | Novagen |
| TG1 | Host for cloning | Novagen |
| BTH101 | Host for the bacterial two-hybrid system | Novagen |
| ***C. glutamicum*** |  |  |
| ATCC13032 | Wild-type *C. glutamicum*, Nal^r^ | Lab stock |
| WT(pXMJ19) | Wild-type *C. glutamicum* harboring pXMJ19, Nal^r^, Cm^r^ | This study |
| Δ*eccD* | *eccD* gene deleted in *C. glutamicum*, Nal^r^ | This study |
| Δ*eccD*(pXMJ19) | Δ*eccD* harboring pXMJ19, Nal^r^, Cm^r^ | This study |
| Δ*eccD*(pXMJ19-*eccD*-*vsvg*) | Δ*eccD* harboring pXMJ19-*eccD*-*vsvg*, Nal^r^, Cm^r^ | This study |
| WT(pEC-XK99E, pXMJ19-*exsI*-*vsvg*) | Wild-type *C. glutamicum* harboring pEC-XK99E and pXMJ19-*exsI*-*vsvg*, Nal^r^, Cm^r^, Km^r^ | This study |
| Δ*eccD*(pEC-XK99E, pXMJ19-*exsI*-*vsvg*) | Δ*eccD* harboring pEC-XK99E and pXMJ19-*exsI*-*vsvg*, Nal^r^, Cm^r^, Km^r^ | This study |
| Δ*eccD*(pEC-XK99E-*eccD*, pXMJ19-*exsI*-*vsvg*) | Δ*eccD* harboring pEC-XK99E-*eccD* and pXMJ19-*exsI*-*vsvg*, Nal^r^, Cm^r^, Km^r^ | This study |
| Δ*eccC* | *eccC* gene deleted in *C. glutamicum*, Nal^r^ | This study |
| Δ*eccC*(pXMJ19) | Δ*eccC* harboring plasmid pXMJ19, Nal^r^, Cm^r^ | This study |
| Δ*eccC*(pXMJ19-*eccC*-*vsvg*) | Δ*eccC* harboring pXMJ19-*eccC*-*vsvg*, Nal^r^, Cm^r^ | This study |
| Δ*exsI* | *exsI* gene deleted in *C. glutamicum*, Nal^r^ | This study |
| Δ*exsI*(pXMJ19) | Δ*exsI* harboring pXMJ19, Nal^r^, Cm^r^ | This study |
| Δ*exsI*(pXMJ19-*exsI*-*vsvg*) | Δ*exsI* harboring pXMJ19-*exsI*-*vsvg*, Nal^r^, Cm^r^ | This study |
| Δ*exsI*(pXMJ19-*exsI^M12A^*-*vsvg*) | Δ*exsI* harboring pXMJ19-*exsI^M12A^*-*vsvg*, Nal^r^, Cm^r^ | This study |
| Δ*exsI*(pXMJ19-*MSMEG_1538*-*vsvg*) | Δ*exsI* harboring pXMJ19- *MSMEG_1538*-*vsvg*, Nal^r^, Cm^r^ | This study |
| Δ*exsI*(pXMJ19-*Rv3445c*-*vsvg*) | Δ*exsI* harboring pXMJ19-*Rv3445c*-*vsvg*, Nal^r^, Cm^r^ | This study |
| Δ*exsI*(pXMJ19-*jk1748*-*vsvg*) | Δ*exsI* harboring pXMJ19-*jk1748*-*vsvg*, Nal^r^, Cm^r^ | This study |
| Δ*exsI*(pXMJ19-*DIP0558*-*vsvg*) | Δ*exsI* harboring pXMJ19-*DIP0558*-*vsvg*, Nal^r^, Cm^r^ | This study |
| Δ*fur* | *fur* gene deleted in *C. glutamicum*, Nal^r^ | This study |
| Δ*fur*(pXMJ19) | Δ*fur* harboring pXMJ19, Nal^r^, Cm^r^ | This study |
| Δ*fur*(pXMJ19-*fur*) | Δ*fur* harboring pXMJ19-*fur*, Nal^r^, Cm^r^ | This study |
| Δ*exiR* | *exiR* gene deleted in *C. glutamicum*, Nal^r^ | This study |
| Δ*exiR*(pXMJ19) | Δ*exiR* harboring pXMJ19, Nal^r^, Cm^r^ | This study |
| Δ*exiR*(pXMJ19-*exiR*-*vsvg*) | Δ*exiR* harboring pXMJ19-*exiR*-*vsvg*, Nal^r^, Cm^r^ | This study |
| WT(pXMJ19-*gfp-exiR*) | Wild-type *C. glutamicum* harboring pXMJ19-*gfp-exiR*, Nal^r^, Cm^r^ | This study |
| ***B. thailandensis*** |  |  |
| E264 | Wild-type *B. thailandensis* (ATCC 700388); environmental isolate from Thailand, Str^r^ | Lab stock |
| ***M. smegmatis*** |  |  |
| mc^2^155 | Wild-type *M. smegmatis* | (1) |
| mc^2^155(pMV261) | Wild-type *M. smegmatis* harboring pMV261, Km^r^ | This study |
| Δ*exsI^ms^* | *exsI^ms^* gene deleted in *M. smegmatis*, Km^r^ | This study |
| Δ*exsI^ms^*(pMV261) | Δ*exsI^ms^* harboring pMV261, Km^r^ | This study |
| Δ*exsI^ms^*(pMV261-*exsI^ms^*) | Δ*exsI^ms^* harboring pMV261-*exsI^ms^*, Km^r^ | This study |
| **Mice** | | |
| Mouse: C57BL/6 | BEIJING HFG BIOSCIENCE CO.,LTD | N/A |
| Mouse: *S100a9*^–/–^ | Cyagen Inc., China | N/A |
| **Cells** | | |
| Mouse: Raw264.7 cells | ATCC | Cat# TIB-71 |
| **Plasmid** | | |
| pXMJ19 | Shuttle vector, Cm^r^ | (2) |
| pXMJ19-*eccD-vsvg* | pXMJ19 carrying *eccD-vsvg* coding region, Cm^r^ | This study |
| pXMJ19-*eccC-vsvg* | pXMJ19 carrying *eccC-vsvg* coding region, Cm^r^ | This study |
| pXMJ19-*exsI-vsvg* | pXMJ19 carrying *exsI*-*vsvg* coding region, Cm^r^ | This study |
| pXMJ19-*fur* | pXMJ19 carrying *fur* coding region, Cm^r^ | This study |
| pXMJ19-*exiR-vsvg* | pXMJ19 carrying *exiR-vsvg* coding region, Cm^r^ | This study |
| pXMJ19-*Rv3445c-vsvg* | pXMJ19 carrying *Rv3445c-vsvg* coding region, Cm^r^ | This study |
| pXMJ19-*MSMEG_1538-vsvg* | pXMJ19 carrying *MSMEG_1538-vsvg* coding region, Cm^r^ | This study |
| pXMJ19-*jk1748-vsvg* | pXMJ19 carrying *jk1748-vsvg* coding region, Cm^r^ | This study |
| pXMJ19-*DIP0558-vsvg* | pXMJ19 carrying *DIP0558-vsvg* coding region, Cm^r^ | This study |
| pXMJ19-*gfp*-*exiR* | pXMJ19 carrying *gfp*-*exiR* coding region, Cm^r^ | This study |
| pEC-XK99E | Shuttle vector, Km^r^ | Lab stock |
| pEC-XK99E-*eccD* | pEC-XK99E carrying *eccD* coding region, Km^r^ | This study |
| pET28a | Expression vector with N-terminal hexahistidine affinity tag, Km^r^ | Novagen |
| pET28a-*exsI* | pET28a carrying *exsI* coding region, Km^r^ | This study |
| pET28a-*exsI^M12A^* | pET28a carrying *exsI^M12A^* coding region, Km^r^ | This study |
| pET28a-*exsI^E8A^* | pET28a carrying *exsI^E8A^* coding region, Km^r^ | This study |
| pET28a-*exsI^E8A/M12A^* | pET28a carrying *exsI^E8A/M12A^* coding region, Km^r^ | This study |
| pET28a-*exsI*-*Cys* | pET28a carrying *exsI*-*Cys* coding region, Km^r^ | This study |
| pET28a-*fur* | pET28a carrying *fur* coding region, Km^r^ | This study |
| pGEX-6p-1 | Expression vector with N-terminal GST tag, Amp^r^ | Novagen |
| pGEX-6p-1-*exsI* | pGEX-6p-1 carrying *exsI* coding region, Amp^r^ | This study |
| pET21aHMT | Expression vector with N-terminal MBP tag, Amp^r^ | Lab stock |
| pET21aHMT-*Rv3445c* | pET21aHMT carrying *Rv3445c* coding region, Amp^r^ | This study |
| pET21aHMT-*MSMEG_1538* | pET21aHMT carrying *MSMEG_1538* coding region, Amp^r^ | This study |
| pET21aHMT-*jk1748* | pET21aHMT carrying *jk1748* coding region, Amp^r^ | This study |
| pET21aHMT-*DIP0558* | pET21aHMT carrying *DIP0558* coding region, Amp^r^ | This study |
| pUT18C | ColE1 origin of replication encoding CyaA_225-399_; Amp^r^ | (3) |
| pUT18C-*exsI* | *exsI* in pUT18C | This study |
| pKT25 | p15A origin of replication encoding CyaA_1-224_; Km^r^ | (3) |
| pKT25-*exiR* | *exiR* in pKT25 | This study |
| pK18*mobsacB* | Suicide plasmid carrying *sacB* for selecting double crossover in *C. glutamicum*, Km^r^ | (4) |
| pK18*mobsacB*-Δ*eccC* | Construct used for in-frame deletion of *eccC*, Km^r^ | This study |
| pK18*mobsacB*-Δ*eccD* | Construct used for in-frame deletion of *eccD*, Km^r^ | This study |
| pK18*mobsacB*-Δ*exsI* | Construct used for in-frame deletion of *exsI*, Km^r^ | This study |
| pK18*mobsacB*-Δ*exiR* | Construct used for in-frame deletion of *exiR*, Km^r^ | This study |
| pK18*mobsacB*-Δ*fur* | Construct used for in-frame deletion of *fur*, Km^r^ | This study |
| pMV261 | Shuttle vector; replicates extrachomosomally in both *E. coli* and *mycobacterium*. ( oriM; oriE; Kn^r^ ) | (1) |
| pMV261-*exsI^ms^* | pMV261 carrying *exsI^ms^* coding region, Km^r^ | This study |
| pYC1240 | Shuttle vector; Hyg^r^ | (1) |
| pYC1240-*sgrna* | pYC1240 carrying *sgrna* coding region, Hyg^r^ | This study |

Nal^r^, Cm^r^, Km^r^, Str^r^, Amp^r^ and Hyg^r^ represent resistance to nalidixic acid, chloramphenicol, kanamycin, streptomycin, ampicillin and hygromycin, respectively.

**Table S2. Primers used in this study.**

| **Primers** | **5’-3’ sequence** | **Function** |
| --- | --- | --- |
| D*eccD*-F1 | GGAAACAGCTATGACCATGATTACGAATTCACCACAAGGTTGCTGGATCAG(*Eco*RI) | To generate pK18*mobsacB*-Δ*eccD* |
| D*eccD*-R1 | TTCTAAAATTTCATCCAAAATGTCC |  |
| D*eccD*-F2 | GGACATTTTGGATGAAATTTTAGAAAATCACTGTGTCTTGCAGCTAGTCT |  |
| D*eccD*-R2 | CGTTGTAAAACGACGGCCAGTGCCAAGCTTCAGGATAAACGGTAGATCCAACTG(*Hin*dIII) |  |
| *eccD*-F1 | TTCACACAGGAAACAGAATTAATTAAGCTTAAAGGAGGACAACCTTGGTTATTTTGGCAATCGATAAT(*Hin*dIII) | To generate pXMJ19-*eccD-vsvg* |
| *eccD*-R1 | GCTGAATTCGAGCTCGGTACCCGGGGATCCTTATTTTCCTAATCTATTCATTTCAATATCTGTATATCCACCGAATCCAATATCAAG(*Bam*HI) |  |
| *eccD*-F2 | AAACAGACCATGGAATTCGAGCTCGGTACCAAAGGAGGACAACCTTGGCAATCGATAATGCGC(*Kpn*I) | To generate pEC-XK99E-*eccD* |
| *eccD*-R2 | ACAGCCAAGCTTGCATGCCTGCAGGTCGACTTATCCACCGAATCCAATATCAAG(*Sal*I) |  |
| D*eccC*-F1 | AGGAAACAGCTATGACATGATTACGAATTCTTCTAAAATTTCATCCAAAATGTCC(*Eco*RI) | To generate pK18*mobsacB*-Δ*eccC* |
| D*eccC*-R1 | TGATCTGTCGCTTTCGCCC |  |
| D*eccC*-F2 | GGGCGAAAGCGACAGATCAGCCCGACATTTATTTAGTTATCGAT |  |
| D*eccC*-R2 | CGTTGTAAAACGACGGCCAGTGCCAAGCTTTCACGCCTTTGGAAGCAAA(*Hin*dIII) |  |
| *eccC*-F | TTCACACAGGAAACAGAATTAATTAAGCTTAAAGGAGGACAACCGTGGAGCGACAAGAACAATCAC(*Hin*dIII) | To generate pXMJ19-*eccC-vsvg* |
| *eccC*-R | GCTGAATTCGAGCTCGGTACCCGGGGATCCTCATTTTCCTAATCTATTCATTTCAATATCTGTATATGAGTCATCACCTATGCGTG(*Bam*HI) |  |
| D*exsI*-F1 | GGAAACAGCTATGACCATGATTACGAATTCGATCAAGTGAAACAAATAGCAGGT(*Eco*RI) | To generate pK18*mobsacB*-Δ*exsI* |
| D*exsI*-R1 | AGCATCACATCGGATTCTGTC |  |
| D*exsI*-F2 | GACAGAATCCGATGTGATGCTCGCTGGACTCCCACTGTAAAAC |  |
| D*exsI*-R2 | CGTTGTAAAACGACGGCCAGTGCCAAGCTTACGTACCACTTACGGGTGATGTC(*Hin*dIII) |  |
| *exsI*-F1 | TTCACACAGGAAACAGAATTAATTAAGCTTAAAGGAGGACAACCATGTCAAATCTTTTCAGGACAGAATC(*Hin*dIII) | To generate pXMJ19-*exsI-vsvg* |
| *exsI*-R1 | GCTGAATTCGAGCTCGGTACCCGGGGATCCTTATTTTCCTAATCTATTCATTTCAATATCTGTATACAGTGGGAGTCCAGCGC(*Bam*HI) |  |
| *exsI*-F2 | ACTGGTGGACAGCAAATGGGTCGCGGATCCATGTCAAATCTTTTCAGGACAGAATC(*Bam*HI) | To generate pET28a-*exsI* and pET28a-*exsI-Cys* |
| *exsI*-R2 | GTGGTGGTGCTCGAGTGCGGCCGCAAGCTTTTACAGTGGGAGTCCAGCGC(*Hin*dIII) |  |
| *exsI*-R | GTGGTGGTGCTCGAGTGCGGCCGCAAGCTTTTA**GCA**CAGTGGGAGTCCAGCGC(*Hin*dIII) |  |
| *exsI*-F3 | GAAGTTCTGTTCCAGGGGCCCCTGGGATCCATGTCAAATCTTTTCAGGACAGA(*Bam*HI) | To generate pGEX-6p-1-*exsI* |
| *exsI*-R3 | GTCAGTCACGATGCGGCCGCTCGAGTCGACTTACAGTGGGAGTCCAGCGC(*Sal*I) |  |
| *exsI*-F4 | ACTGGTGGACAGCAAATGGGTCGCGGATCCATGTCAAATCTTTTCAGGACAGAATCCGATGTG**GCG**CTCGCG(*Bam*HI) | To generate pET28a-*exsI^M12A^* |
| *exsI*-R4 | GTGGTGGTGCTCGAGTGCGGCCGCAAGCTTTTACAGTGGGAGTCCAGCGCCGTCGCCAGCACC(*Hin*dIII) |  |
| *exsI*-F5 | ACTGGTGGACAGCAAATGGGTCGCGGATCCATGTCAAATCTTTTCAGGACA**GCA**TCCGAT(*Bam*HI) | To generate pET28a-*exsI^E8A^* and pET28a-*exsI^E8A/M12A^* |
| *exsI*-R5 | GTGGTGGTGCTCGAGTGCGGCCGCAAGCTTTTACAGTGGGAGTCCAGCGCCGTC(*Hin*dIII) |  |
| *exsI*-F6 | CGCGGATCCCATGTCAAATCTTTTCAGGACAGAATC(*Bam*HI) | To generate pUT18C-*exsI* |
| *exsI*-R6 | CGGGGTACCTTACAGTGGGAGTCCAGCGC(*Kpn*I) |  |
| D*fur*-F1 | AGGAAACAGCTATGACATGATTACGAATTCTAACGATGTTGCTTCCGCTG(*Eco*RI) | To generate pK18*mobsacB*-Δ*fur* |
| D*fur*-R1 | CGGGGCAGAGCCTTGG |  |
| D*fur*-F2 | CCAAGGCTCTGCCCCGGGACTTTGCGCTGATTGTAAG |  |
| D*fur*-R2 | CGTTGTAAAACGACGGCCAGTGCCAAGCTTCGCTGATCCGTGGCCTAC(*Hin*dIII) |  |
| *fur*-F1 | TTCACACAGGAAACAGAATTAATTAAGCTTAAAGGAGGACAACCGTGGGTATCAATCGCATCAGC(*Hin*dIII) | To generate pXMJ19-*fur* |
| *fur*-R1 | GCTGAATTCGAGCTCGGTACCCGGGGATCCCTACGTAACTTTTTCCTTACAATCAGC(*Bam*HI) |  |
| *fur*-F2 | ACTGGTGGACAGCAAATGGGTCGCGGATCCGTGGGTATCAATCGCATCAGC(*Bam*HI) | To generate pET28a-*fur* |
| *fur*-R2 | GTGGTGGTGCTCGAGTGCGGCCGCAAGCTTCTACGTAACTTTTTCCTTACAATCAGC(*Hin*dIII) |  |
| D*exiR*-F1 | AGGAAACAGCTATGACATGATTACGAATTCGATCGGCTATTGGAGGTGGA(*Eco*RI) | To generate pK18*mobsacB*-Δ*exiR* |
| D*exiR*-R1 | TTCAAGGCTGCTCATAGTCCG |  |
| D*exiR*-F2 | CGGACTATGAGCAGCCTTGAAACAAAACAGCCTGGACTAAGGTG |  |
| D*exiR*-R2 | CGTTGTAAAACGACGGCCAGTGCCAAGCTTCTGGTGTCGGTGCCTTCTACA(*Hin*dIII) |  |
| *exiR*-F1 | TTCACACAGGAAACAGAATTAATTAAGCTTAAAGGAGGACAACCATGAGCAGCCTTGAAGGGTTT(*Hin*dIII) | To generate pXMJ19-*exiR-vsvg* |
| *exiR*-R1 | GCTGAATTCGAGCTCGGTACCCGGGGATCCTTATTTTCCTAATCTATTCATTTCAATATCTGTATAGTCCAGGCTGTTTTGTCCC(*Bam*HI) |  |
| *exiR*-F2 | CGCGGATCCATGAGCAGCCTTGAAGGGTTT(*Bam*HI) | To generate pKT25-*exiR* |
| *exiR*-R2 | CGGGGTACCTTAGTCCAGGCTGTTTTGTCCC(*Kpn*I) |  |
| *exsI^ms^*-UP-F | TCGACGTGTTGCAGCGAG | To generate Δ*exsI^ms^* |
| *exsI^ms^*-UP-R | ACAGTGGTCATCACGTCGAAAT |  |
| *exsI^ms^*-DOWN-F | ATTTCGACGTGATGACCACTGTACCCTCTGACCCGCAGAC |  |
| *exsI^ms^*-DOWN-R | ATTTGTGGGATCAGACGCC |  |
| *exsI^ms^*-F1 | CCGGAATTCATGTCGACACCGCTGGG(*Eco*RI) | To generate pMV261-*exsI^ms^* |
| *exsI^ms^*-R1 | CCCAAGCTTTCAGAGGGTCTCGCCGAC(*Hin*dIII) |  |
| *MSMEG_1538*-F2 | CCGGAATTCATGTCGACACCGCTGGG(*Eco*RI) | To generate pET21aHMT-*MSMEG_1538* |
| *MSMEG_1538*-R2 | CCGCTCGAGTCAGAGGGTCTCGCCGAC(*Xho*I) |  |
| *MSMEG_1538*-F3 | TTCACACAGGAAACAGAATTAATTAAGCTTAAAGGAGGACAACCATGTCGACACCGCTGGG(*Hin*dIII) | To generate pXMJ19-*MSMEG_1538-vsvg* |
| *MSMEG_1538*-R3 | GCTGAATTCGAGCTCGGTACCCGGGGATCCTCATTTTCCTAATCTATTCATTTCAATATCTGTATAGAGGGTCTCGCCGAC(*Bam*HI) |  |
| *Rv3445c*-F1 | TTCACACAGGAAACAGAATTAATTAAGCTTAAAGGAGGACAACCGTGAGCACACCGAACACGC(*Hin*dIII) | To generate pXMJ19-*Rv3445c-vsvg* |
| *Rv3445c*-R1 | GCTGAATTCGAGCTCGGTACCCGGGGATCCCTATTTTCCTAATCTATTCATTTCAATATCTGTATATAGGTCGCCGCCGG(*Bam*HI) |  |
| *Rv3445c*-F2 | CCGGAATTCGTGAGCACACCGAACACGC(*Eco*RI) | To generate pET21aHMT-*Rv3445c* |
| *Rv3445c*-R2 | CCGCTCGAGCTATAGGTCGCCGCCGG(*Xho*I) |  |
| *jk1748*-F1 | TTCACACAGGAAACAGAATTAATTAAGCTTAAAGGAGGACAACCATGAGCTTTAAAACAGATGTCAGCA(*Hin*dIII) | To generate pXMJ19-*jk1748-vsvg* |
| *jk1748*-R1 | GCTGAATTCGAGCTCGGTACCCGGGGATCCTTATTTTCCTAATCTATTCATTTCAATATCTGTATAAGCGTTGAAAGCTGCTGC(*Bam*HI) |  |
| *jk1748*-F2 | CCGGAATTCATGAGCTTTAAAACAGATGTCAGCA(*Eco*RI) | To generate pET21aHMT-*jk1748* |
| *jk1748*-R2 | CCGCTCGAGTTAAGCGTTGAAAGCTGCTGC(*Xho*I) |  |
| *DIP0558*-F1 | TTCACACAGGAAACAGAATTAATTAAGCTTAAAGGAGGACAACCATGTCTCAAGGTTTTAAGACCGAAG(*Hin*dIII) | To generate pXMJ19-*DIP0558-vsvg* |
| *DIP0558*-R1 | GCTGAATTCGAGCTCGGTACCCGGGGATCCTTATTTTCCTAATCTATTCATTTCAATATCTGTATACAGCGCGAGCCCTTG(*Bam*HI) |  |
| *DIP0558*-F2 | CCGGAATTCATGTCTCAAGGTTTTAAGACCGAAG(*Eco*RI) | To generate pET21aHMT-*DIP0558* |
| *DIP0558*-R2 | CCGCTCGAGTTACAGCGCGAGCCCTTG(*Xho*I) |  |
| *sgrna*-F | (*Bpm*I)ATCGACGTGATGACCACTGTGGCAGGAA(*Hin*dIII) | To generate pYC1240-*sgrna* |
| *sgrna*-R | (*Bpm*I)AGCTTTCCTGCCACAGTGGTCATCACGTCGATCT(*Hin*dIII) |  |
| *gfp*-*exiR*-UP-F | TTCACACAGGAAACAGAATTAATTAAGCTTAAAGGAGGACAACCATGGTGAGCAAGGGCGAG(*Hin*dIII) | To generate pXMJ19-*gfp-exiR* |
| *gfp*-*exiR*-UP-R | CTTGTACAGCTCGTCCATGC |  |
| *gfp-exiR*-DOWN-F | GCATGGACGAGCTGTACAAGATGAGCAGCCTTGAAGGGTTT |  |
| *gfp*-*exiR*-DOWN-R | GCTGAATTCGAGCTCGGTACCCGGGGATCCTTAGTCCAGGCTGTTTTGTCCC(*Bam*HI) |  |
| *T7SS*-*eccB*-F | GCCCGCAATAATGTTGAACG | EMSA |
| *T7SS*-*eccB*-R | CCATGTATTCCCCCAGCTTTAG |  |
| Q*16S*-F | AGAACCTTACCTGGGCTTGA | qRT-PCR to detect bacterial gene expression |
| Q*16S*-R | CGCTCGTTGCGGGACTTA |  |
| Q*eccB*-F | TGATTGCGGAAGGAAATGG |  |
| Q*eccB*-R | GAGCAGCTCAGTGGTCAGGTAT |  |
| Q*mycP*-F | AGGGCACAGGAAGTAGAAGC |  |
| Q*mycP*-R | CAGACGTGGATGCAGGGA |  |
| Q*eccD*-F | GCGTGAGCATCCGCATAG |  |
| Q*eccD*-R | GGGCTTCCAAGTCCCTTT |  |
| Q*eccC*-F | GGGAGGAACCCAAAGAGG |  |
| Q*eccC*-R | CCACCACAAATAGCCCAGT |  |
| Q*exsI*-F | TTTTCAGGACAGAATCCGATGT |  |
| Q*exsI*-R | GGCGTGCTGATGAGTTCC |  |
| Q*exiR*-F | CCCCATTCCTTCGCATCT |  |
| Q*exiR*-R | AATCCGACGGCTTTCCAC |  |
| *Ferroportin*-F | TGGAACTCTATGGAAACAGCCT | qRT-PCR to detect mouse gene expression |
| *Ferroportin*-R | GGCATTCTTATCCACCCAGT |  |
| *Lcn2*-F | ATGTCACCTCCATCCTGGTCAG |  |
| *Lcn2*-R | GCCACTTGCACATTGTAGCTCTG |  |
| *S100a8*-F | CAAGGAAATCACCATGCCCTCTA |  |
| *S100a8*-R | ACCATCGCAAGGAACTCCTCGA |  |
| *S100a9*-F | TGGTGGAAGCACAGTTGGCAAC |  |
| *S100a9*-R | CAGCATCATACACTCCTCAAAGC |  |
| *Cxcl1*-F | TGCACCCAAACCGAAGTCAT |  |
| *Cxcl1*-R | TTGTCAGAAGCCAGCGTTCAC |  |

*Underlined sites indicate restriction enzyme cutting sites added for cloning. Letters in boldface denote the mutation sites in overlap PCR for site-directed mutagenesis.

## **Table S3. Reagents and resources used in this study.**

| **Reagents** | **Source** | **Identifier** |
| --- | --- | --- |
| **Molecular Reagent** | | |
| Hydrogen peroxide solution 3% | Sigma-Aldrich | 88597, CAS: 7722-84-1 |
| CM-H2DCFDA | Invitrogen | C6827 |
| HPF | Invitrogen | H36004 |
| Alexa Fluor 488 | Thermo Fisher Scientific | A10254 |
| SYBR Safe DNA Gal Stain | Invitrogen | S33102 |
| *PerfectStart* Green qPCR SuperMix | TransGen Biotech | AQ601 |
| 2 × *ApexHF* FS PCR Master Mix | Accurate Biology | AG12202 |
| QuickCut HindIII | TaKaRa | 1615 |
| QuickCut BamHI | TaKaRa | 1605 |
| QuickCut EcoRI | TaKaRa | 1611 |
| QuickCut XhoI | TaKaRa | 1635 |
| QuickCut KpnI | TaKaRa | 1618 |
| *Bpm*I | Biolabs (NEB) | R0565S |
| 2 × Taq Master Mix | Novoprotein | E005 |
| QuickBlock Blocking Buffer for Western Blot | Beyotime | P0252 |
| QuickBlock Primary Antibody Dilution Buffer for Western Blot | Beyotime | P0256 |
| BugBuster Protein Extraction Reagent | Novagen | 3243372 |
| Trans2K Plus II DNA Marker | TransGen Biotech | BM121 |
| Blue Plus II Protein Marker (14-120 kDa) | TransGen Biotech | DM111-02 |
| **Medium and solution** | | |
| Dulbecco's Modified Eagle Medium (DMEM) High glucose | GIBCO | 11965-084 |
| Phosphate-Buffered Saline (PBS) | Beyotime | C0221A |
| Penicillin-Streptomycin Solution (100X) | Beyotime | C0222 |
| Fetal Bovine Serum, FBS | Byotime | C0251 |
| Trypsin-EDTA Solution with phenol red | Byotime | C0203 |
| 7H9 Broth | Hopebio | HB9233 |
| 7H10 Agar | Hopebio | HB6270 |
| Brain Heart Infusion Broth | Solarbio | LA0360 |
| 4% Paraformaldehyde Fix Solution | Beyotime | P0099 |
| Albumin Dextrose Catalase (ADC) Supplement | Hopebio | HB9233a |
| Oleic Albumin Dextrose Catalase (OADC) Supplement | Hopebio | HB6271a |
| Mice | | |
| Mouse: C57BL/6 | Beijing Vital River Laboratory Animal Technology | N/A |
| Mouse: *S100a9^–/–^* | Cyagen | N/A |
| **Antibodies** | | |
| Mouse monoclonal anti-RNAP | Santa Cruz Biotechnology | sc-56766 |
| Mouse monoclonal anti-VSV-G (F-6) | Santa Cruz Biotechnology | sc-365019 |
| His-Tag Mouse Monoclonal Antibody | ABWAYS | AB0002 |
| GST-Tag Mouse Monoclonal Antibody | ABWAYS | AB0003 |
| Peroxidase AffiniPure Goat Anti-Mouse IgG (H+L) | DIYIBIO | DY60203 |
| Peroxidase AffiniPure Goat Anti-Rabbit IgG (H+L) | DIYIBIO | DY60202 |
| **Commercial Assays** | | |
| Universal DNA Purification Kit | TIANGEN | DP214-02 |
| TIANprep Mini Plasmid Kit | TIANGEN | DP103-03 |
| EasyScript One-Step gDNA Removal and cDNA Synthesis SuperMix | TransGen Biotech | AE311-02 |
| RNAprep pure Cell/Bacteria Kit | TIANGEN | DP430 |
| RNAprep Pure Tissue Kit | TIANGEN | DP431 |
| Pierce Sliver Stain Kit | Thermo Fisher Scientific | 24612 |
| TIANamp Bacteria DNA Kit | TIANGEN | DP302 |
| Seamless Cloning Kit | Beyotime | D7010M |
| DNA Ligation Kit | Accurate Biology | AG11801 |
| Pierce ECL Western Blotting Substrate | Invitrogen | 32209 |
| Calprotectin ELISA kit | SHANGHAI HUDING BIOTECHNOLOGY | DB871-Mu |
| **Other** | | |
| Mice diet AIN-76A | Research Diets Inc. | D10001 |
| Graphpad Prism 8.0 software | GraphPad | https://www.graphpad.com/ |
| DNAman 8.0 sofeware | Lynnon Biosoft | https://www.lynnon.com/ |
| MEGA 11.0 software | MEGA | https://www.megasoftware.net/ |
| Alphafold3 | Alphafold | https://alphafoldserver.com/ |
| AutoDock Vina 1.2.5 | AutoDock | https://github.com/ccsb-scripps/AutoDock-Vina |
| PyMOL | Schrödinger | https://pymol.org/ |

**REFERENCES**

1. Yan M Y, Yan H Q, Ren G X, Zhao J P, Guo X P, Sun Y C. 2017. CRISPR-Cas12a-Assisted Recombineering in Bacteria*.* Appl Environ Microbiol 83. <http://doi.org/10.1128/AEM.00947-17>

2. Jakob K, Satorhelyi P, Lange C, Wendisch V F, Silakowski B, Scherer S, Neuhaus K. 2007. Gene expression analysis of *Corynebacterium glutamicum* subjected to long-term lactic acid adaptation*.* Journal of Bacteriology 189:5582-5590. <http://doi.org/10.1128/Jb.00082-07>

3. Karimova G, Pidoux J, Ullmann A, Ladant D. 1998. A bacterial two-hybrid system based on a reconstituted signal transduction pathway*.* Proc Natl Acad Sci U S A 95:5752-6. <http://doi.org/10.1073/pnas.95.10.5752>

4. Schäfer A, Tauch A, Jäger W, Kalinowski J, Thierbach G, Pühler A. 1994. Small mobilizable multi-purpose cloning vectors derived from the *Escherichia coli* plasmids pK18 and pK19: selection of defined deletions in the chromosome of *Corynebacterium glutamicum.* Gene 145:69-73. <http://doi.org/10.1016/0378-1119(94)90324-7>
